# Targeting virulence factors Psl and PcrV restores host defense in bronchiectasis and cystic fibrosis models

**DOI:** 10.1172/JCI205523

**Published:** 2026-09-22

**Authors:** Wanhai Qin, Wayne Brailsford, Stacey Cromer Berman, Christina S. Thornton, Antonio DiGiandomenico, Paul Kubes

**Affiliations:** 1 Calvin, Phoebe, and Joan Snyder Institute for Chronic Diseases, Cumming School of Medicine, and; 2 Department of Physiology and Pharmacology, Cumming School of Medicine, University of Calgary, Calgary, Alberta, Canada.; 3 Respiratory and Immunology, BioPharmaceuticals R&D, AstraZeneca, Gothenburg, Sweden.; 4 Infectious Disease, BioPharmaceuticals R&D, AstraZeneca, Gaithersburg, Maryland, USA.; 5 Division of Respiratory Medicine, Department of Medicine, Cumming School of Medicine, University of Calgary, Calgary, Alberta, Canada.; 6 Department of Biomedical and Molecular Science, Queen’s University, Kingston, Ontario, Canada.

**Keywords:** Immunology, Infectious disease, Microbiology, Cellular immune response, Innate immunity, Mouse models

## Abstract

Chronic *Pseudomonas aeruginosa* infection is a central driver of bronchiectasis and contributes to progressive lung decline in patients with cystic fibrosis (CF), even in the era of CF transmembrane conductance regulator (CFTR) modulators. A major limitation in the field is that conventional mouse models fail to develop persistent, biofilm-associated lung infection, restricting mechanistic studies and preclinical evaluation. Here, we establish clinically relevant infection models by combining CF-like mouse strains (βENaC-overexpressing and CFTR-deficient mice) with agarose bead–embedded *P*. *aeruginosa* that forms persistent, tobramycin-refractory biofilms. Using intravital lung microscopy, we show that alveolar macrophages initially responded by surrounding biofilms but progressively dissociated from the biofilms during persistent infection. Neutrophils were also recruited but failed to clear bacteria. Administration of gremubamab (MEDI3902), a bispecific antibody targeting the virulence factors Psl and PcrV, preserved alveolar macrophages in persistent biofilm infection, restored bacterial sensing and phagocytosis, limited excessive neutrophilic inflammation, and significantly improved bacterial clearance and survival. Together, these findings establish a clinically relevant persistent *P*. *aeruginosa* infection model and highlight gremubamab as a promising virulence-targeted therapy to potentially overcome bacterial immune evasion while restoring host defense in mouse models of bronchiectasis and CF.

## Abstract 2

Targeting key bacterial virulence factors PcrV and Psl restores immune defenses, reduces persistent *P.aeruginosa* infection, and improves survival in mouse models of cystic fibrosis and bronchiectasis.

## Introduction

Persistent *Pseudomonas aeruginosa* lung colonization remains difficult to eradicate as bacteria adapt to the inflamed respiratory tract and organize into aggregates and biofilm-like communities that resist both immune clearance and antimicrobial therapy. This is especially evident in chronic suppurative lung diseases, such as cystic fibrosis (CF) and non-CF bronchiectasis, where persistent *P*. *aeruginosa* infection is linked to exacerbations and mortality (1–3). Although highly effective CF transmembrane conductance regulator (CFTR) modulators have transformed CF care by improving ion transport, mucus hydration, and clinical outcomes (4–8), longitudinal studies show that respiratory infection often persists despite these benefits (9–12). Accordingly, a central unanswered question is how lung host defense fails once *P*. *aeruginosa* transitions from planktonic growth to persistent aggregate-associated infection.

Alveolar macrophages are the dominant resident phagocytes of the distal lung and provide frontline defense at the epithelial surface (13, 14). Using lung intravital microscopy, we previously showed that after planktonic *P*. *aeruginosa* challenge, alveolar macrophages rather than neutrophils dominate early bacterial sensing and phagocytosis in the alveolar space (15). Whether this defense program remains effective during persistent aggregate-associated infection is unknown. Although CF and bronchiectasis are fundamentally airway-centered diseases, distal lung compartments are not exempt from injury in advanced disease, and alveolar inflammation has been described in CF lungs (16, 17). However, the specific mechanisms by which alveolar macrophage–mediated host defense succeeds or fails against persistent *P*. *aeruginosa* aggregates in the distal lung microenvironment remain a fundamental knowledge gap.

Investigating these immune dynamics in vivo requires experimental systems that recapitulate the complexities of persistent infection and structurally abnormal lungs. Agarose bead–embedded *P. aeruginosa* provides a well-established platform to generate localized, persistent bacterial aggregates that model features of persistent infection (18–20). In parallel, CFTR-mutant and βENaC-overexpressing mice capture complementary aspects of CF pathophysiology, including defective ion transport and airway surface dehydration (21, 22). Understanding host-pathogen interactions within these environments is critical, as current management of chronic *P*. *aeruginosa* relies heavily on antipseudomonal antibiotics, which are frequently limited by bacterial tolerance and acquired resistance (23). Consequently, virulence-targeted strategies have emerged as an alternative approach to restore host immune recognition rather than direct bactericidal measures. Key targets include Psl, an exopolysaccharide that promotes aggregation and shields bacteria from phagocytosis, and PcrV, an essential component of the type III secretion system mediating host-cell toxicity (24, 25). The bispecific monoclonal antibody gremubamab (MEDI3902) was specifically designed to couple bacterial opsonization with the inhibition of type III secretion by targeting both Psl and PcrV (26–28). We previously demonstrated that this bispecific antibody, upon binding to Psl on *P. aeruginosa*, promotes neutrophil recognition and phagocytosis of the bacteria. Importantly, bacterial uptake delivers the antibody into the phagolysosome, where it can effectively neutralize PcrV at the site where the type III secretion system is most active (24, 29). Despite these observations, this unique antibody has not been assessed in persistent biofilm lung infections.

To directly visualize these host-pathogen interactions and determine if virulence-targeted therapies can overcome immune evasion, we combined lung intravital microscopy with persistent bead-based infection in murine models that manifest certain defects also found in suppurative lung diseases, such as bronchiectasis and CF. We sought to characterize the specific mechanisms by which aggregate-associated bacteria evade resident host defenses by tracking real-time cellular behaviors in the distal lung. Finally, we further expand this model to test whether dual targeting of Psl and PcrV with gremubamab can restore effective bacterial clearance.

## Results

### Disrupted lung immune landscapes and alveolar macrophage dysfunction in CF models.

Both βENaC-overexpressing mice, which develop airway surface dehydration and mucus obstruction (30), and CFTR^ΔF508^ mice, which carry the most common CF-causing CFTR mutation in lungs (31), have been widely used as models of CF; however, each model has important limitations. To combine both sets of deficiencies in these models, also found in patients with CF and bronchiectasis, we crossed the ENaC and CFTR mice and generated CFTR/ENaC mice, which carry both the CFTR^ΔF508^ mutation and βENaC overexpression. As previously documented (30), ENaC mice exhibit mucus overproduction, as do the CFTR/ENaC mice, while the CFTR mutation alone does not increase mucus production (Figure 1, A and B) but has other pronounced deficiencies (discussed later). To characterize the immune landscape in the alveolar spaces of these mice, we collected BALF from adult mice for flow cytometric analysis. Immune cells were significantly elevated in the alveolar spaces of both ENaC and CFTR/ENaC mice, primarily due to increased neutrophils (from less than 100 to more than 30,000 cells in the CFTR/ENaC group) (Figure 1, C–E). Eosinophils also increased but to a lesser degree than neutrophils. A negligible increase in B cells was noted (Figure 1, F and G). Although AM numbers were comparable across all mouse strains (Figure 1D), AMs from ENaC and CFTR/ENaC mice exhibited a subtle but significantly lower expression of the AM identity markers Siglec-F and CD11c, alongside a small but significant increase in the activation marker CD11b, suggesting some fundamental changes in AMs in these 2 strains of mice. In contrast, AMs from CFTR mice showed no changes in the expression of these surface markers compared with WT mice (Figure 1, H–J, and Supplemental Figure 2; supplemental material available online with this article; https://doi.org/10.1172/JCI205523DS1).

AMs are the predominant patrolling immune cells in healthy WT alveoli, where their efficient movement is essential for effective clearance of pathogens and debris (15). To investigate AM behavior within the distinct alveolar microenvironments of these CF models, AM motility was quantified using intravital imaging (Figure 1K). Compared with WT mice, AM movement, displacement, and total migration distance over 2 hours were significantly reduced in CFTR/ENaC mice, a phenotype prevalent in the CFTR and much less obvious in the ENaC single-mutant mice. (Figure 1L). Collectively, these findings demonstrate that ENaC mice had increased mucus production while the CFTR^–/–^ mice had impairment in AM behavior. The CFTR/ENaC mice exhibited both deficiencies.

### Planktonic P. aeruginosa fails to establish chronic lung infection in all strains of mice.

When fluorescent planktonic *P*. *aeruginosa* was injected into the lungs of WT mice, where AMs and neutrophils were fluorescent, intravital imaging revealed that AMs were able to crawl to and efficiently phagocytose essentially all the bacteria within 2 hours, while neutrophils, which reside in the capillaries of the lung, remained in this compartment and did not contribute to the bacterial eradication (Supplemental Video 1). When we delivered 1 million bacteria oropharyngeally, we observed an increase in neutrophil recruitment, and the majority of bacteria were gone by 24 hours and completely eradicated in all mice, except a few remnant bacteria left in ENaC/CFTR mice at 48 hours (Figure 2, A, B, and D), suggesting this strain had the greatest difficulty clearing *P*. *aeruginosa*. This aligned with significantly more neutrophil counts (Figure 2, C and E). Although CFTR/ENaC mice did show impaired capacity to eradicate *P*. *aeruginosa*, they eventually did clear the infection, failing to develop chronic infections. Clearly, planktonic delivery of *P*. *aeruginosa* failed to recapitulate the chronic disease seen in patients with bronchiectasis and CF. In an attempt to develop a chronic model of *P*. *aeruginosa*, we increased the infectious dose, but mice began succumbing by day 2 (Figure 2F). Notably, a subset of mice survived the high-dose infection, and these mice harbored variable bacterial burdens in the lungs at the experimental endpoint (Figure 2G). Thus, in these CF mouse models using planktonic infection, bacteria were either cleared rapidly or expanded uncontrollably at higher doses, leading to early mortality. Together, these findings demonstrate that planktonic delivery of *P*. *aeruginosa* fails to recapitulate the persistent infection characteristic of bronchiectasis and CF.

### Induction of persistent infection with P. aeruginosa beads.

Next, we embedded *P*. *aeruginosa* into agarose beads and imaged their location after oropharyngeal inoculation into mouse lungs. These bacterial beads dispersed evenly throughout the lungs such that similar numbers of beads were seen near the pleural surface and deeper within the lung (Figure 3, A and B). Notably, most agarose beads remained intact, releasing minimal individual bacteria by day 3 postinfection (Figure 3, A and B). The bacterial beads persisted in lungs at least until day 7 postinfection, where we could now see microcolonies emanating from the beads and disseminating throughout the lung, a chronic feature of human disease (Figure 3C). To determine biofilm formation on the *P*. *aeruginosa* agarose beads and within infected lungs, Psl, a major biofilm component, was stained using an anti-Psl–specific antibody. Psl was observed on the surface of agarose beads harboring WT PA01 but not Psl-deficient *P*. *aeruginosa* in vitro (Figure 3D) and was visualized surrounding the agarose beads within lung tissues at 5 days postinfection (Figure 3, E and F), confirming biofilm formation both in vitro and in vivo.

Clinically, tobramycin is an important antipseudomonal antibiotic widely used for eradication and chronic suppressive therapy in CF and non-CF bronchiectasis (32, 33). While highly effective in complete clearance of bacteria during acute, 6-hour infection (Supplemental Figure 4, A and B), tobramycin treatment significantly reduced but failed to completely clear bacterial burden from lung tissues infected with agarose bead–embedded bacteria even at 72 hours (Figure 3, G and H). In this model, bacterial load decreased over 7 days, but significant bacterial persistence was still observed at 7 days (Figure 3, C and I) and at 14 days (not shown). Mice experienced initial weight loss over 2 days, followed by gradual recovery to baseline by day 7 (Figure 3J). Infection led to significant increase of lung weight at day 1 and lasted until 7 days (the latest time point examined) (Figure 3K). Importantly, no mice reached humane endpoint or succumbed to infection when inoculated with agarose beads containing 0.5 million CFU of *P*. *aeruginosa*. AMs efficiently phagocytosed free *P*. *aeruginosa* when administered planktonically during the first 2 hours of infection such that within 2 hours the majority of *P*. *aeruginosa* had disappeared (Supplemental Figure 4C and Supplemental Video 1). By contrast, imaging agarose beads at day 7 for 2 hours revealed free microcolonies emanating from the beads with no AMs or neutrophils phagocytosing the bacteria. These findings suggest that the recruitment of both AMs and neutrophils to sites of bacterial persistence is largely lost by day 7 in the chronic infection (Figure 3L and Supplemental Video 2).

Collectively, these data indicate that infection of mice with *P*. *aeruginosa* agarose beads successfully establishes a chronic infection characterized by persistent bacterial colonization, biofilm formation, antibiotic resistance, and immune evasion.

### AM dynamics during P. aeruginosa chronic infection.

AMs are the predominant immune sentinel cells patrolling the alveoli to protect lung tissue from infections (13, 14). A previous study has suggested that the patrolling efficiency of AMs plays a crucial role in eliminating pathogens from the lungs (15). To characterize the dynamic behavior of AMs during chronic *P*. *aeruginosa* infection, we employed intravital microscopy to image AMs at multiple time points following infection with *P*. *aeruginosa* agarose beads. Soon after infection, AMs rapidly moved toward the bacterial beads, often touching the beads (Figure 4A and Supplemental Video 3). Six hours postinfection, bacterial beads were partially surrounded by a limited number of AMs, while additional AMs from greater distances continued to migrate toward the beads (Figure 4B and Supplemental Video 4). By 24 hours postinfection, AMs had tightly encircled the bacterial beads, but no further recruitment of distant AMs was observed. Notably, AMs ceased their patrolling activity and were all stationary at this time point (Figure 4C and Supplemental Video 5). By day 3 postinfection, the number of AMs around the beads was markedly reduced. Furthermore, the AM ring that had formed on day 1 was no longer apparent, and no AMs were observed migrating toward the bacterial beads at this time (Figure 4D and Supplemental Video 6). At 7 days postinfection, AMs’ movement had halted, and they failed to effectively phagocytose *P*. *aeruginosa* bacterial microcolonies that grew out of the beads (Figure 4E and Supplemental Video 7).

CD11b and AM canonical marker Siglec-F have been widely used to distinguish resident AMs from monocyte-derived macrophages (MoMs) by flow cytometry (34). To further validate this gating strategy, *Ms4a3 Cre Rosa TdT* mice, which report on MoMs, were given CellVue Claret Far Red dye prior to infection (to label all AMs). Then the 2 macrophage populations were tracked throughout the 7 days of infection. As shown in Supplemental Figure 5, the Siglec-F–high population consisted exclusively of PKH26-positive cells, suggesting no contribution from MoMs. In contrast, the CD11b-high population was predominantly tdTomato (Ms4a3) positive and largely lacked PKH26 labeling, consistent with newly recruited monocyte-derived AMs. Therefore, the 2 macrophage populations can be distinguished based on their surface expression of Siglec-F and CD11b. Using this approach, we observed a significant decrease in AM numbers within the first 24 hours postinfection, followed by a gradual return to baseline levels by day 7 (Figure 4F). Additionally, the surface expression of Siglec-F significantly decreased within the first 3 days postinfection but began to recover by day 7 (Figure 4G). Conversely, CD11b, indicative of AM activation, progressively increased postinfection, peaking on day 3 and remaining elevated until day 7 (Figure 4H). It is well established that monocytes are recruited to the lung during infection and differentiate into MoMs. In line with this, we observed a significant increase in MoM numbers starting 24 hours after infection (Figure 4I).

To assess the localization of the MoMs within the infectious nidus, we used the *Ms4a3 CreERT2 Rosa TdT* mice, in which the Ms4a3^TdT^ reporter mediates tdTomato expression upon tamoxifen administration, thereby excluding any MoMs that were in the lung prior to infection (35). At 5 days postinfection, we found many tdTomato-traced cells in the lung, but most of them were not found in direct contact with either the bacterial beads or individual microcolonies (Figure 4J). The very large red cells are the MoMs whereas the much smaller red cells that are also labeled with tdTomato in the reporter system are neutrophils. At day 5 neither cell type was interacting with the bacteria, suggesting that MoMs were not phagocytosing the bacteria, which is consistent with a previous report indicating that monocyte recruitment to the lung does not significantly influence *P*. *aeruginosa* colonization or bacterial clearance (36). To assess the role of AMs in controlling infection, AMs were depleted using intratracheal clodronate liposomes prior to infection (Figure 4K). This treatment reduced AM numbers by approximately 85% (Figure 4L), resulting in a significantly increased bacterial burden at day 3 postinfection and significantly elevated mortality (Figure 4, M and N), thereby demonstrating a critical role for AMs in bacterial clearance. Since this approach did not affect the monocytes or MoMs, this further supports the view that these cells were not able to clear the persistent *P*. *aeruginosa* biofilm. These observations suggest that AMs rapidly respond to *P*. *aeruginosa* beads by migrating toward them shortly after infection and becoming activated, accompanied by changes in their cellular identity, and play a significant role in preventing the bacteria from killing the host. Intravital imaging further revealed that AM antimicrobial behavior does tend to wane with time during the course of the persistent biofilm infection, ultimately leading to reduced pathogen uptake efficiency at later stages.

### Neutrophil dynamics during P. aeruginosa chronic infection.

Neutrophils are rapidly recruited to sites of bacterial infection, where they serve as a critical first line of defense in the innate immune response (37). To visualize the dynamics of neutrophils during *P. aeruginosa* chronic infection, we imaged neutrophil behavior by IVM at multiple time points in Catchup mice, whose neutrophils are fluorescently labeled by tdTomato expression under the control of the Ly6G promoter (38), after *P*. *aeruginosa* bead administration. Neutrophils were not significantly recruited in the first 3 hours after infection (Figure 5A and Supplemental Video 8). Between 3 and 6 hours, neutrophils rapidly recruited and accumulated around bacterial beads, tightly surrounding them by 4 hours postinfection, with continued recruitment at 6 hours (Figure 5B and Supplemental Video 9). By 24 hours after infection, neutrophils entirely enveloped the beads, with no further new recruitment observed (Figure 5C and Supplemental Video 10). At day 3, neutrophils still colocalized with bacterial beads (Figure 5D and Supplemental Video 11). By day 7, using IVM imaging, we saw the majority of neutrophils were now located within the vasculature and not in the parenchyma (Supplemental Video 12). They were no longer associating with bacterial beads or microcolonies (Figure 5E and Supplemental Video 12). Consistent with the intravital imaging, flow cytometric analysis confirmed rapid neutrophil recruitment in the lung tissues, which peaked at 24 hours (10 million neutrophils), declined 5-fold by day 3 (to 2 million), and further decreased (to 200,000) by day 7 postinfection (Figure 5F).

### The roles of Psl and PcrV in P. aeruginosa chronic infection and gremubamab-mediated protection in mice.

Psl and PcrV are 2 key virulence factors conserved in the majority of *P*. *aeruginosa* strains, including those chronic isolates from patients with CF or bronchiectasis (28, 39). To validate the role of Psl and PcrV in infection, mice were infected for 3 days, a period characterized by peak inflammation and maximal body weight loss, with agarose beads embedded with WT, *Psl*-deficient (Δ*Psl*), or *PcrV*-deficient (Δ*PcrV*) *P*. *aeruginosa* (Figure 6A). Mice infected with WT or Δ*Psl* strains experienced body weight loss over the first 3 days. In contrast, mice infected with Δ*PcrV* bacteria exhibited significantly less body weight loss and began regaining weight by day 3 (Figure 6, B and C). Bacterial burden was significantly reduced in lungs infected with Δ*PcrV* agarose beads but was unchanged in those infected with Δ*Psl* beads, compared with WT-infected controls (Figure 6D). Lung weights followed a similar trend, with lower weights in Δ*PcrV*- and Δ*Psl*-infected mice, though not statistically significant for the Δ*Psl* group (Figure 6E). Flow cytometric analysis revealed total immune cell and neutrophil counts were lower, whereas AMs were significantly more abundant in lungs infected with Δ*PcrV* beads (Figure 6, F–H). These findings suggest that PcrV plays a crucial role, while Psl has a more subtle role in persistent *P*. *aeruginosa* biofilm infection, perhaps because other molecules such as Pel may compensate (16). Nevertheless, attaching an antibody that could be recognized by myeloid cells to the surface of the biofilm could help these immune cells recognize and internalize this pathogen.

To evaluate the efficacy of targeting Psl or PcrV in chronic infection in CF mice, anti-PcrV antibody (αPcrV), anti-Psl antibody (αPsL), an isotype control antibody (Iso α), as well as the bispecific antibody (for both Psl and PcrV), gremubamab, were given, and then *P*. *aeruginosa* agarose beads were instilled (Figure 6I). Both αPcrV and gremubamab, but not αPsL, significantly reduced bacterial burden and neutrophil infiltration compared with controls (Figure 6, J and K). Additionally, gremubamab-treated mice — and to a lesser extent, αPcrV-treated mice — showed increased numbers of AMs accompanied by higher expression of AM identity markers Siglec-F and CD11c (Figure 6, L–N), suggesting that neutralizing PcrV provided some protection to these AMs. AM activation, reflected by CD11b expression, was enhanced in αPsL- or gremubamab-treated mice (Figure 6O). Neutrophil activation (indicated by CD11b expression) was also significantly elevated in αPsL- and gremubamab-treated mice (Figure 6P). To further evaluate the effects of gremubamab treatment on AM and neutrophil behavior, we imaged infected lungs at 5 days postinfection (Figure 6Q). Consistent with previous findings (Figure 3), few AMs and neutrophils were observed surrounding bacterial beads at this later time point, and more bacterial microcolonies were now present in the isotype antibody–treated lungs. The majority of the microcolonies were not phagocytosed by either AMs or neutrophils (Figure 6, R and S). However, in gremubamab-treated lungs, more AMs and fewer microcolonies emanating from the beads were detected, with a greater proportion of bacteria associated with AMs compared with control antibody–treated lungs (Figure 6S). In a separate set of experiments, a lethal dose (2 × 10^6^ CFU) of *P*. *aeruginosa* beads was administered, and treatment with αPcrV or gremubamab significantly improved mouse survival following infection with this higher dose of *P*. *aeruginosa* (Figure 6T). Thus, neutralizing PcrV or dual targeting of Psl and PcrV with gremubamab effectively protects mice from persistent *P*. *aeruginosa* bead infection by modulating AM and neutrophil responses and helping eradicate bacteria.

### CFTR deficiency compromises defense against chronic P. aeruginosa infection, and gremubamab confers protection.

Although CFTR mutations underlie CF, CFTR-knockout (CFTR) mice typically do not show increased susceptibility to acute *P*. *aeruginosa* infection as seen in human patients (Figure 2). Since AMs normally migrate toward bacterial beads within the first few hours (Figure 4), we hypothesized that AMs in CFTR mice are functionally impaired in this process, as their migration in naive CFTR mice is significantly slower than that observed in WT controls. To test this, mice were infected with *P*. *aeruginosa* agarose beads for 6 hours, after which their lungs were imaged (Figure 7A). Consistent with this hypothesis, fewer AMs were found surrounding the beads in the lungs of CFTR mice (Figure 7, B and C). To further evaluate pulmonary immune defense during persistent infection, CFTR mice were infected with *P*. *aeruginosa* agarose beads for 3 days (Figure 7D). Compared with WT controls, CFTR mice exhibited significantly increased lung weight and bacterial burden (Figure 7, E and F). Using higher concentrations of bacterial biofilm showed reduced survival rates in CFTR mice (Figure 7G), indicating impaired pulmonary defense against persistent infection.

Given gremubamab’s potential efficacy against chronic *P*. *aeruginosa* infection in WT mice, its protective capability was further assessed in CFTR mice. CFTR mice received gremubamab treatment 24 hours prior to infection with *P*. *aeruginosa* agarose beads (Figure 7H). Gremubamab treatment significantly mitigated body weight loss and prevented an increase in lung weight among CFTR mice by day 3 postinfection (Figure 7, I and J). It reduced the accumulation of total immune cells and neutrophils in the lungs while preserving AM populations (Figure 7, K–M). Additionally, gremubamab enhanced neutrophil activation, evidenced by increased CD11b expression, and maintained AM identity as it preserved Siglec-F and CD11c levels (Figure 7, N and O). Moreover, gremubamab significantly decreased lung bacterial burden relative to isotype antibody-treated controls (Figure 7P). Therefore, these data demonstrate that CFTR mice exhibit defects in controlling chronic *P*. *aeruginosa* infection and highlight the prophylactic potential of gremubamab in a CFTR-deficient context.

### Gremubamab mitigates persistent P. aeruginosa infection in ENaC mice.

Next, ENaC mice were infected with *P*. *aeruginosa* agarose beads (Figure 8A). Following infection, ENaC mice exhibited pronounced body weight loss, increased lung weight, and altered immune cell infiltration compared with WT controls (Figure 8, B–D). A significant neutrophil accumulation and a significant depletion of AMs were noted (Figure 8, E and F), indicating substantial immune dysregulation during persistent biofilm infection. Consistent with this, bacterial burden was significantly higher in ENaC mice than in WT counterparts (Figure 8G). When ENaC mice received gremubamab treatment and *P*. *aeruginosa* agarose beads (Figure 8H), gremubamab significantly reduced bacterial burden and neutrophil accumulation in the lung (Figure 8, I and J) while restoring AM numbers (Figure 8K). In a separate set of experiments, gremubamab treatment significantly improved survival following a high-dose *P*. *aeruginosa* infection in the ENaC mice (Figure 8L).

To further mimic the cystic fibrotic lung microenvironment, CFTR/ENaC mice were chronically infected with *P*. *aeruginosa* beads (Figure 9A). Similar to ENaC or CFTR mice, CFTR/ENaC mice exhibited impaired pulmonary defense, characterized by elevated bacterial burden (Figure 9B), increased total immune cell influx, and reduced AM numbers (Figure 9, C and D). Notably, AMs from CFTR/ENaC mice displayed decreased expression of the identity marker Siglec-F and elevated expression of the activation marker CD11b (Figure 9, E and F). These mice also exhibited greater neutrophil accumulation, accompanied by a reduction in the activation marker CD11b on neutrophil surfaces (Figure 9, G and H). Consistent with the impaired immune defense, CFTR/ENaC mice showed higher mortality following high-dose *P*. *aeruginosa* infection (Figure 9I). Administration of gremubamab 24 hours prior to infection (Figure 9J) significantly improved outcomes in CFTR/ENaC mice, including a reduced bacterial burden, decreased lung weight, attenuated immune cell infiltration and neutrophil accumulation, and preserved AM populations (Figure 9, K–O). Collectively, these findings demonstrate that CFTR/ENaC mice exhibit impaired immune responses to chronic *P*. *aeruginosa* infection, effectively modeling CF-associated lung pathology. Importantly, gremubamab treatment restored pulmonary host defense and ameliorated persistent infection in all 3 strains of mice having various lung impairments.

### Therapeutic administration of gremubamab after establishment of P. aeruginosa infection reduces bacterial burden and improves survival.

To assess the therapeutic efficacy of gremubamab in a clinically relevant setting, WT mice were infected with *P*. *aeruginosa*–containing agarose beads and treated 24 hours later, thereby modeling the initiation of therapy after infection had already been established. Mice received control antibody, tobramycin, gremubamab, or a combination of gremubamab and tobramycin (Figure 10A). Analysis of lung bacterial burden on day 3 postinfection revealed that treatment with gremubamab alone or in combination with tobramycin significantly reduced bacterial loads compared with control antibody–treated mice (Figure 10B). In contrast, tobramycin alone did not significantly improve bacterial clearance. Furthermore, the addition of tobramycin did not provide further reduction in bacterial burden beyond that achieved by gremubamab alone (Figure 10B). Despite the reduction in bacterial burden, total lung leukocyte numbers, neutrophils, and AMs were not significantly altered among treatment groups (Figure 10, C–E). This might be expected, since immune cell numbers were already changed when treatment was initiated. To determine whether gremubamab retains therapeutic efficacy in a CF-relevant setting, CFTR mice were infected with *P*. *aeruginosa* beads and treated with gremubamab 24 hours after infection (Figure 10F). Therapeutic administration of gremubamab significantly reduced lung bacterial loads compared with control-treated mice at day 3 postinfection (Figure 10G). Total leukocyte, neutrophil, and AM numbers showed a trend toward improvement in gremubamab-treated mice, though these differences did not reach statistical significance (Figure 10H). However, therapeutic administration of gremubamab significantly improved survival compared with control-treated mice when the CFTR mice were challenged with a higher inoculum of *P*. *aeruginosa* beads (Figure 10I). Together, these findings demonstrate that gremubamab remains effective when administered after infection has been established, reducing pulmonary bacterial burden and improving survival in both WT and CFTR mice.

## Discussion

Chronic *P*. *aeruginosa* colonization remains a central driver of lung function decline and early mortality in cystic fibrosis, even with the use of CFTR modulators. Despite improved airway hydration and mucociliary clearance, most individuals continue to harbor *P*. *aeruginosa*, reflecting the pathogen’s exceptional capacity for persistence and resistance. Biofilm formation, metabolic adaptation, and antibiotic tolerance together render *P*. *aeruginosa* nearly impossible to eradicate once established (23, 40). Repeated antibiotic therapy offers only transient control and often selects for multidrug-resistant strains (41), underscoring the urgent need for alternative approaches that target virulence and restore host defense.

In this study, we show that gremubamab, a bispecific monoclonal antibody directed against the *P*. *aeruginosa* virulence factors Psl and PcrV, protects against a persistent biofilm infection in mouse models with features of both CF and bronchiectasis. Using CFTR, β-ENaC, and CFTR/ENaC transgenic mice, which recapitulate airway mucus obstruction, chronic inflammation, and impaired immune function, we demonstrate that gremubamab significantly reduces bacterial burden, limits neutrophil infiltration, preserves AMs, and improves survival. These effects were consistent across both acute infection (not shown) and our persistent biofilm model established using *P*. *aeruginosa* agarose beads. Furthermore, therapeutic administration of gremubamab after infection establishment in WT and CFTR mice also significantly enhanced bacterial clearance and improved survival, supporting its efficacy in both prophylactic and postinfection/colonization.

CFTR^ΔF508^ mice have been widely used to model cystic fibrosis; however, these mice do not develop the hallmark features of human CF lung disease or consistently exhibit increased susceptibility to *P*. *aeruginosa* infection. Nevertheless, CFTR-deficient mice provide a valuable model for investigating the consequences of CFTR dysfunction on innate immune responses, host defense, and susceptibility to persistent *P*. *aeruginosa* infection. In contrast, βENaC-overexpressing mice develop airway surface dehydration, impaired mucociliary clearance, mucus obstruction, and chronic airway inflammation, key pathological features prevalent in CF and other muco-obstructive lung diseases (21). The CFTR/βENaC double-mutant model combines these complementary pathological features, enabling the assessment of how defective CFTR signaling and mucus obstruction interact to influence infection susceptibility, disease progression, and therapeutic responses. Together, these models provide a comprehensive framework for investigating host-pathogen interactions and evaluating novel therapeutic strategies across multiple facets of lung pathology associated with a persistent lung infection. We observed that AMs particularly in the CFTR mice exhibit reduced motility and altered surface marker expression, indicating defects in patrolling and phagocytic capacity. These abnormalities likely contribute to the chronic inflammation and impaired bacterial clearance characteristic of CF airways. Although these mice display compromised responses to persistent infection, planktonic *P*. *aeruginosa* is typically eliminated within 2 days, suggesting that in all these mouse strains myeloid cells still have some capacity to move and eradicate bacteria.

To better model chronic infection, we established an agarose bead infection model that induces localized, biofilm-like *P*. *aeruginosa* aggregates within the lung. These infections persisted for up to 2 weeks and exhibited tolerance to tobramycin, recapitulating key clinical features of chronic *P*. *aeruginosa* infection. Taking advantage of intravital imaging, we found that during persistent infection, AMs, the resident sentinels of the lung, migrate toward bacterial beads within the first few hours, followed by the rapid recruitment of neutrophils to the infection site. While the AMs were critical for restraining bacterial growth and preventing mortality, their effectiveness waned, leading to massive neutrophil influx, increased inflammation, and edema formation. Although the agarose bead model has been widely used to study chronic infection, previous investigations have primarily employed CFTR-deficient or WT mice (42–44) and the innate immune cell–bacterial interactions have not been imaged. Consistent with these earlier studies, we found that CFTR-deficient mice were more susceptible to chronic *P*. *aeruginosa* infection. Building on this established model, we combined persistent biofilm infection with lung IVM to investigate the cellular mechanisms underlying impaired host defense in CF lungs. Using this approach, we identified a potentially previously unrecognized defect in AM behavior, characterized by reduced migration toward *P*. *aeruginosa*–containing agarose beads during the early stages of infection. Combined with a significant amount of AM death, this almost certainly leaves the host susceptible to ongoing and disseminating infection. By extending these studies to βENaC and CFTR/βENaC mice, which recapitulate the muco-obstructive environment of lungs of CF and bronchiectasis, we characterized immune responses across multiple disease-relevant models. These studies revealed a remarkable capacity of *P*. *aeruginosa* to evade both neutrophils and macrophages and to establish microcolonies that remain largely inaccessible to these immune cells, consistent with our previous observations regarding Psl-mediated immune cloaking (26, 29).

As a key component of the type III secretion system (T3SS), PcrV enables *P*. *aeruginosa* to inject cytotoxic effectors into host cells, promoting inflammation and impairing immune defense (25). Loss of PcrV function, by either genetic ablation or antibody-mediated neutralization, significantly reduces bacterial burden and inflammation. In contrast, although biofilm-associated mechanisms contribute to infection persistence, targeting the biofilm polysaccharide Psl alone had only modest effects. Dual neutralization of PcrV and Psl with gremubamab effectively integrated these mechanisms, overcoming the constraints of single-target approaches. Furthermore, our previous investigation suggests that incorporating anti-Psl and anti-PcrV within the same molecule is more effective in acute bloodstream infections than administering anti-PcrV and anti-Psl monoclonal antibodies in combination (26, 29). While gremubamab has shown efficacy in acute pneumonia models, its role in chronic infection remained uncertain. In this study, gremubamab significantly reduced bacterial load, attenuated neutrophil-driven inflammation, and preserved AM populations in all these CFTR, ENaC, and CFTR/ENaC CF mouse models. Intravital imaging confirmed improved AM-bacteria interactions after gremubamab administration, suggesting decreased AM death and restored macrophage function. By simultaneously disarming T3SS-dependent cytotoxicity and biofilm-mediated persistence, gremubamab rebalances host-pathogen interactions and restores bacterial clearance. These findings establish gremubamab as a potent antivirulence strategy against chronic *P*. *aeruginosa* and underscore its translational potential in the context of antibiotic resistance. Rather than exerting direct bactericidal pressure, gremubamab neutralizes virulence determinants, potentially synergizing with antibiotics or CFTR modulators. Importantly, both Psl and PcrV are highly conserved among diverse *P*. *aeruginosa* clinical isolates recovered from patients with CF and bronchiectasis (39, 45). Furthermore, clinical isolates obtained from children with persistent infection exhibit increased anti-Psl antibody binding (46), further supporting the relevance of Psl and PcrV as broadly applicable therapeutic targets across diverse *P*. *aeruginosa* populations. Notably, early-phase clinical studies have reported favorable safety profiles and pharmacokinetics of gremubamab, supporting the translational potential of this dual-targeted antibody (27, 47). Moreover, a subsequent phase IIb trial (GREAT-2) in patients with bronchiectasis colonized with *P*. *aeruginosa* yielded encouraging results. In the GREAT-2 study, delivery of gremubamab resulted in a significant reduction of *P*. *aeruginosa* airway bacterial load, decreased exacerbation frequency, and improvements in patient-reported quality of life among patients with bronchiectasis, thereby successfully meeting the study’s primary endpoint (48). Our work highlights some of the mechanisms by which this antibody functions.

Several limitations of this study should be considered. First, although the agarose bead model enables reproducible persistent *P*. *aeruginosa* infection and direct visualization of host-pathogen interactions, the smaller beads used in this study likely deposit preferentially within the distal lung and alveolar compartment rather than the small airways, where infection is primarily initiated and maintained in human CF and bronchiectasis (49). Nevertheless, it is well documented that there is alveolar involvement in end-stage CF, making our study relevant to that aspect of the disease (16). In addition, infections were established using the laboratory strain PAO1, which may not fully capture the phenotypic diversity, adaptive evolution, and persistence mechanisms of CF-adapted clinical isolates (50). Chronic CF isolates frequently exhibit altered virulence factor expression, enhanced biofilm-associated behavior, and increased antimicrobial tolerance (51). Consequently, the relative contribution of individual virulence determinants observed in this study may not be representative of all clinical strains. However, it is worth mentioning that a comprehensive analysis of 913 globally distributed clinical *P*. *aeruginosa* isolates demonstrated that 99% (902/913) retained an intact *pcrV* gene (39). Moreover, other biofilm-associated factors, such as the exopolysaccharide Pel (52), contribute to bacterial persistence and treatment resistance but are not targeted by gremubamab and require a similar strategy but targeting different bacterial virulence factors for some patients. Second, each mouse model captures only selected aspects of CF lung disease. CFTR-deficient mice model CFTR dysfunction but do not develop the mucus-obstructive airway disease characteristic of human CF, whereas βENaC-overexpressing mice model airway dehydration and mucus obstruction but retain normal CFTR function. In addition, the gut-corrected FABP-hCFTR model may express human CFTR in certain myeloid cell populations (53), which could partially influence immune phenotypes. Therefore, these models do not fully recapitulate the airway-centered anatomy or immune microenvironment of human CF and bronchiectatic lung disease. However, the significant impairment in crawling seen in the AMs in the gut-corrected FABP-hCFTR model strongly supports the existence of this phenotype in patients with CF. Finally, neutrophils, resident AMs, and MoMs were not simultaneously visualized in the same animal, limiting our ability to define their spatial relationships around bacterial aggregates. Finally, future studies could assess cytokine production or other transcriptional programs not examined herein, to better understand the full spectrum of inflammation in these model systems. Taken together, these murine models provide a useful platform for mechanistic studies, but caution is warranted when extrapolating directly to human CF and bronchiectatic airway disease.

In conclusion, we established a persistent *P*. *aeruginosa* infection model in CF-like lungs and demonstrated that gremubamab effectively mitigates *P*. *aeruginosa* biofilm infections by simultaneously targeting 2 conserved virulence determinants, Psl and PcrV. This dual mechanism preserves AM function, attenuates neutrophil-driven inflammation, and enhances bacterial clearance in models that closely recapitulate human CF pathology. Collectively, these findings provide compelling evidence supporting further clinical development of bispecific antibody therapy for the management of chronic *P*. *aeruginosa* infection/colonization in CF, bronchiectasis, and other chronic lung diseases.

## Methods

### Sex as a biological variable.

Both male and female mice were used in this study. Sex was not considered as a biological variable.

### Mice.

B6N.Cg-Tg (Scgb1a1-Scnn1b)6608Bouc/J mice (JAX stock 006438) were obtained from Jackson Laboratory and bred with C57BL/6 mice to generate transgenic mice with airway-specific overexpression of epithelial Na^+^ channels (ENaC), here referred to as ENaC mice. Gut-corrected Cftrtm1KthTg(FABPCFTR)1Jaw/Cwr mice (Craig Hodges, Case Western Reserve University, Cleveland, Ohio, USA), here referred to as CFTR mice, carry the *Cftr* F508del mutation but also express human CFTR under the control of the rat fatty acid binding protein promoter (FABP-hCFTR), allowing for intestinal CFTR expression. ENaC mice were crossed with CFTR mice to generate CFTR (homozygous ki)/F508del/βENaC mice (CFTR/ENaC), which harbor the *Cftr* F508del mutation and overexpress ENaC in airway epithelial cells. Rosa26tdTomato (Ai14) mice (JAX stock 007914) were bred in-house and crossed with *Ms4a3*-*CreERT* mice or *Ms4a3Cre* mice (gifts from Florent Ginhoux, Shanghai Jiao Tong University School of Medicine, Shanghai, China) to generate *Ms4a3^ERT^*-tdTomato and *Ms4a3Cre*-tdTomato reporter mice, respectively. Ly6G-Cre/Ai14 (CatchupIVM-red; here referred to as Catchup mice) were provided by Matthias Gunzer (University Duisburg–Essen, Essen, Germany.). All mice were maintained under specific pathogen–free, double-barrier conditions at the University of Calgary and used for experiments at 6–10 weeks of age. Our study examined male and female animals, and similar findings are reported for both sexes.

### Lung IVM.

Intravital lung imaging was performed as previously described (15). In brief, AMs were labeled with PKH26 phagocytic dye (Sigma-Aldrich, PKH26GL) or CellVue Claret phagocytic dye (Sigma-Aldrich, MINCLARET) through oropharyngeal administration 3–5 days before imaging. IVM imaging of AMs, neutrophils, and bacterial beads was performed using a Nikon multichannel spinning-disk confocal microscope. Mice were anesthetized with ketamine (200 mg/kg) and xylazine (10 mg/kg) via intraperitoneal injection and maintained on a 37°C heated pad. The right jugular vein was cannulated for administration of fluorescent antibodies and supplemental anesthetic. The trachea of the mouse was exposed, and a small catheter connecting to a mini rodent ventilator was threaded into the trachea. A ~1.5 cm intercostal incision between 2 ribs was made to expose the lung, and a window was inserted with gentle suction (~20 mm Hg) to stabilize the tissue. Images were acquired using a 10×/0.45 NA objective with 488, 561, and 635 nm laser excitation. Time-lapse *z*-stacks (50 μm depth, 5 μm step size) were collected for up to 3 hours. Macrophage and neutrophil behavior was analyzed using Imaris (Bitplane).

### Bacteria.

WT *P*. *aeruginosa* strain PA01 were cultured in brain heart infusion (BHI) medium. Psl- or PcrV-knockout strains (PAΔPsl and PAΔPcrv, respectively) and green fluorescent protein expressing–strain were generated previously with PAO1 background (29).

### Acute pneumonia.

Acute pneumonia induction and sampling were performed as previously described (54). Briefly, overnight bacterial cultures were subcultured for 4 hours, washed with cold PBS, and resuspended in PBS to an OD_600_ of 0.5. Mice were inoculated oropharyngeally with 1 × 10^6^ CFU in 50 μL PBS after being anesthetized with 3% isoflurane (in O_2_, 2 L/min). For antibody treatment, mice received an intraperitoneal injection of either control mAb (11.4 mg/kg; molar equivalent to 150 kDa IgG) or gremubamab (15 mg/kg; 200 kDa) 24 hours prior to infection. Six hours postinfection, mice were euthanized, and BALF (1.5 mL cold sterile PBS) was collected to assess bacterial burden and quantify immune cell influx by flow cytometry.

### P. aeruginosa agarose bead preparation.

Bacterial beads were generated as previously described (55). Briefly, subcultured *P*. *aeruginosa* were washed with cold PBS, resuspended in PBS, and mixed with 2.25 mL of freshly autoclaved, warm (≈45°C) 1.5% BHI agar. The bacterial-agar suspension was then slowly dispensed, using a 3 mL syringe fitted with a 19-gauge needle, into ice-cold mineral oil containing 0.1% (v/v) Tween-20 under continuous stirring. The mixture was stirred for 20 minutes in an ice bath to allow bead formation. The resulting beads were collected and washed 10 times with cold PBS to remove residual mineral oil. Beads were subsequently filtered through a 100 μm cell strainer to eliminate large aggregates, followed by filtration through a 40 μm strainer to remove smaller beads and free bacteria. The beads retained on the 40 μm strainer were rinsed off and resuspended in cold PBS (Supplemental Figure 3A). The diameter of the bacterial beads was confirmed by microscopy (Supplemental Figure 3, B and C). Bacterial load within the beads was quantified by serial dilution plating. Prepared beads were stored at 4°C for up to 2 days and freshly prepared before each experiment.

### Chronic pneumonia.

Mice were inoculated oropharyngeally with 50 μL of agarose beads containing 0.2 × 10^6^ to 2 × 10^6^ CFU of bacteria suspended in 50 μL PBS. Because WT, CFTR, ENaC, and CFTR/ENaC mice exhibit different susceptibilities to *P*. *aeruginosa*, the infection dose was adjusted for each mouse strain. WT mice were inoculated with 0.5 or 2 × 10^6^ CFU, CFTR mice with 0.2 or 1 × 10^6^ CFU, and ENaC as well as CFTR/ENaC mice with 0.2 or 0.5 × 10^6^ CFU. For direct comparisons between WT and CF mice, comparable CFU doses were used to match the infection burden in CF mice. For antibody treatment, mice received an intraperitoneal injection of a control mAb (11.4 mg/kg; molar equivalent to a 150 kDa IgG), anti-Psl antibody, anti-PcrV antibody, or gremubamab (15 mg/kg; 200 kDa) 24 hours prior to infection. Animals were euthanized at designated points postinfection. To distinguish immune cells within lung tissues from those in circulation, mice were anesthetized with 3% isoflurane (in O_2_, 2 L/min) and injected intravenously with anti-CD45 antibody 3 minutes before euthanasia by cervical dislocation. Lung tissues were then harvested for assessment of bacterial burden and immune cell infiltration.

### Flow cytometric analysis.

BAL fluid samples were processed as previously described (54). Immune cell populations in the BAL fluid were identified based on surface marker expression as follows: AMs (CD45^+^CD64^+^Siglec-F^+^CD11c^+^), neutrophils (CD45^+^Ly6G^+^CD11b^+^), monocytes (CD45^+^Ly6C^+^CD11b^+^), MoMs (CD45^+^CD64^+^Siglec-F^–^Ly6C^lo^CD11b^+^), eosinophils (CD45^+^Siglec-F^+^CD11c^–^CD11b^+^), B cells (CD45^+^CD19^+^), and T cells (CD45^+^CD3^+^). To compare the AM surface marker expression, representative histograms of Siglec-F, CD11c, and CD11b are provided in Supplemental Figure 2. Lung tissues were digested with collagenase and DNase, and immune cell populations within lung tissue (negative for intravenously administered CD45 antibody) were analyzed using the same gating strategy as described above. The representative gating strategy is shown in Supplemental Figure 1. All the antibodies used can be found in Supplemental Table 1.

### Statistics.

The Mann-Whitney *U* test was used for comparisons between 2 groups, whereas Kruskal-Wallis test was used for comparisons among more than 2 groups. All statistical analyses were performed using the GraphPad Prism program. A *P* value of less than 0.05 was considered statistically significant. Data shown in figures as mean ± SEM.

### Study approval.

All experimental procedures were reviewed and approved by the University of Calgary Animal Care Committee of the University of Calgary, Alberta, Canada (protocol no. AC20-0121 and AC24-0127), and conducted in accordance with the Canadian Council on Animal Care guidelines.

### Data availability.

All data are available upon request from the corresponding authors. Values for all data points in graphs are reported in the Supporting Data Values XLS file. Any requests for the reagents and materials used in this study that are not commercially available should be directed to the corresponding authors.

## Manuscript information

### Author contributions.

WQ performed experiments, collected and analyzed data, drafted the manuscript, and organized the experimental design. WB, SCB and CST critically analyzed and formatted the manuscript. AD and PK provided guidance and insight on experimental design, critically analyzed data, and formatted the manuscript.

### AI disclosure.

ChatGPT (OpenAI; GPT-5.2 and subsequent GPT-5 series models) was used between January and October 2026 solely for grammatical correction and language polishing of the manuscript. It was not used for research design, data extraction, data analysis, data interpretation, synthesis of research findings, preparation of figures or graphics, or generation of scientific content.

### Conflict of interest.

AD, WB, and SCB are employees of AstraZeneca and might hold shares in the company.

### Funding.

This study was supported by the following:

- AstraZeneca (partial funding).
- Banting Postdoctoral Fellowship (WQ).
- Eyes-High Postdoctoral Fellowship (WQ).
- AIMS Postdoctoral Fellowship (WQ).
- CIHR (10034311, PK).

## Supplementary Material

Supplemental data

Supplemental video 1

Supplemental video 2

Supplemental video 3

Supplemental video 4

Supplemental video 5

Supplemental video 6

Supplemental video 7

Supplemental video 8

Supplemental video 9

Supplemental video 10

Supplemental video 11

Supplemental video 12

Supporting data values

## Figures and Tables

**Figure 1:**
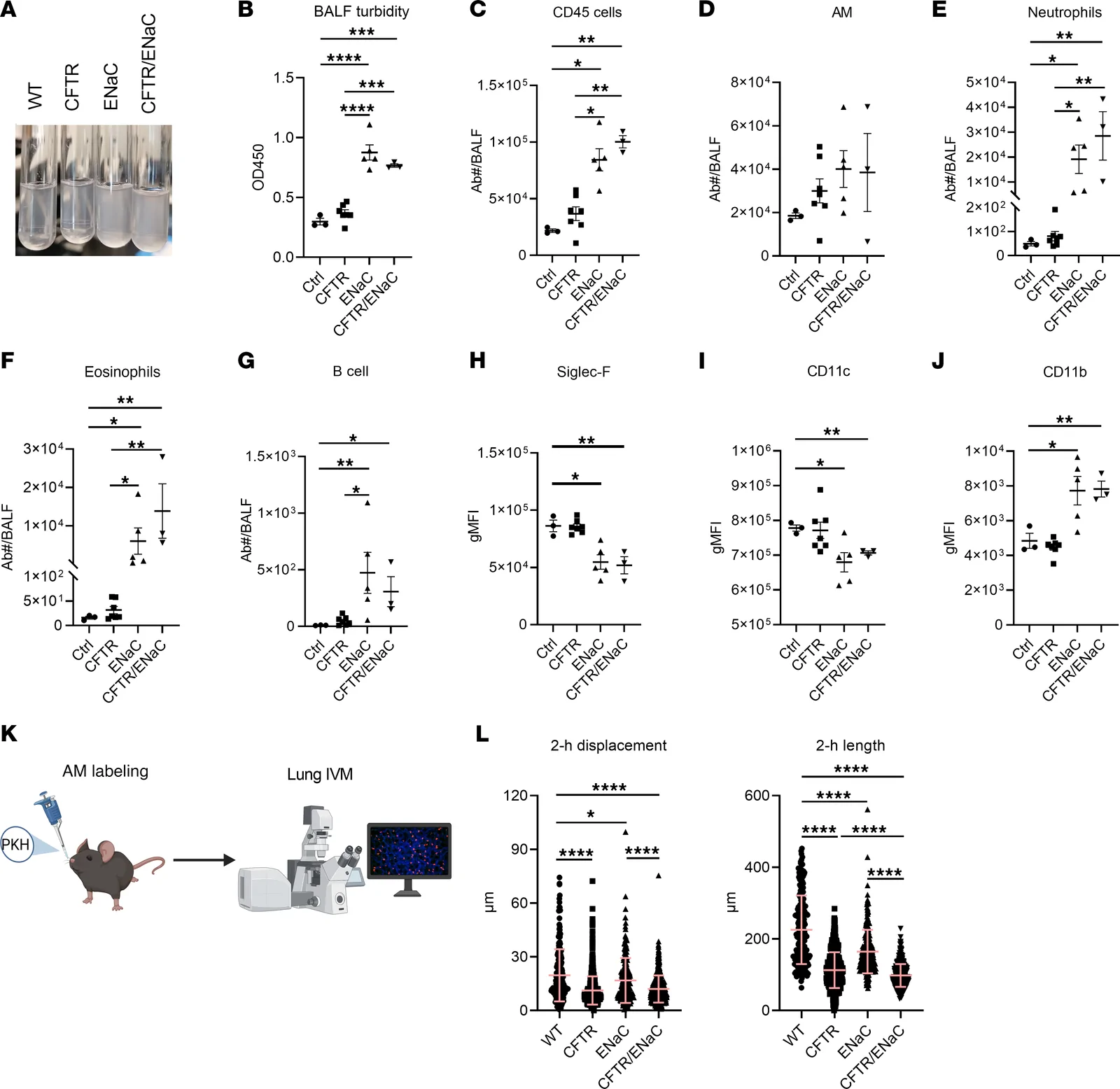
Characterization of alveolar macrophages in wild-type and CF mouse models. (**A**) Representative bronchoalveolar lavage fluid (BALF) samples collected from wild-type (WT) control, CFTR, ENaC, and CFTR/ENaC mice. (**B**) Turbidity of BALF samples. (**C**–**G**) Numbers of total immune cells, alveolar macrophages (AMs), neutrophils, eosinophils, and B cells in the BALF. (**H**–**J**) Surface protein expression of Siglec-F, CD11c, and CD11b on AMs. gMFI, geometric mean fluorescence intensity. (**K**) PKH26 was administered to adult mice to label AMs before lung intravital microscopy (IVM) imaging. (**L**) AM displacement and total migration distance over 2 hours. Data show mean ± SEM with individual data shown as dots. One-way ANOVA with Tukey’s post hoc test and statistical significance: *P < 0.05, **P < 0.01, ***P < 0.001, ****P < 0.0001. Each data point represents an individual mouse from 1 representative experiment except **L**, where each dot represents an AM from 4 mice. Independent experiments yielded similar results and are representative of *N* ≥ 2 experiments. **K** was created in BioRender. Qin, W. (n.d.) https://BioRender.com/yajdzlp.

**Figure 2:**
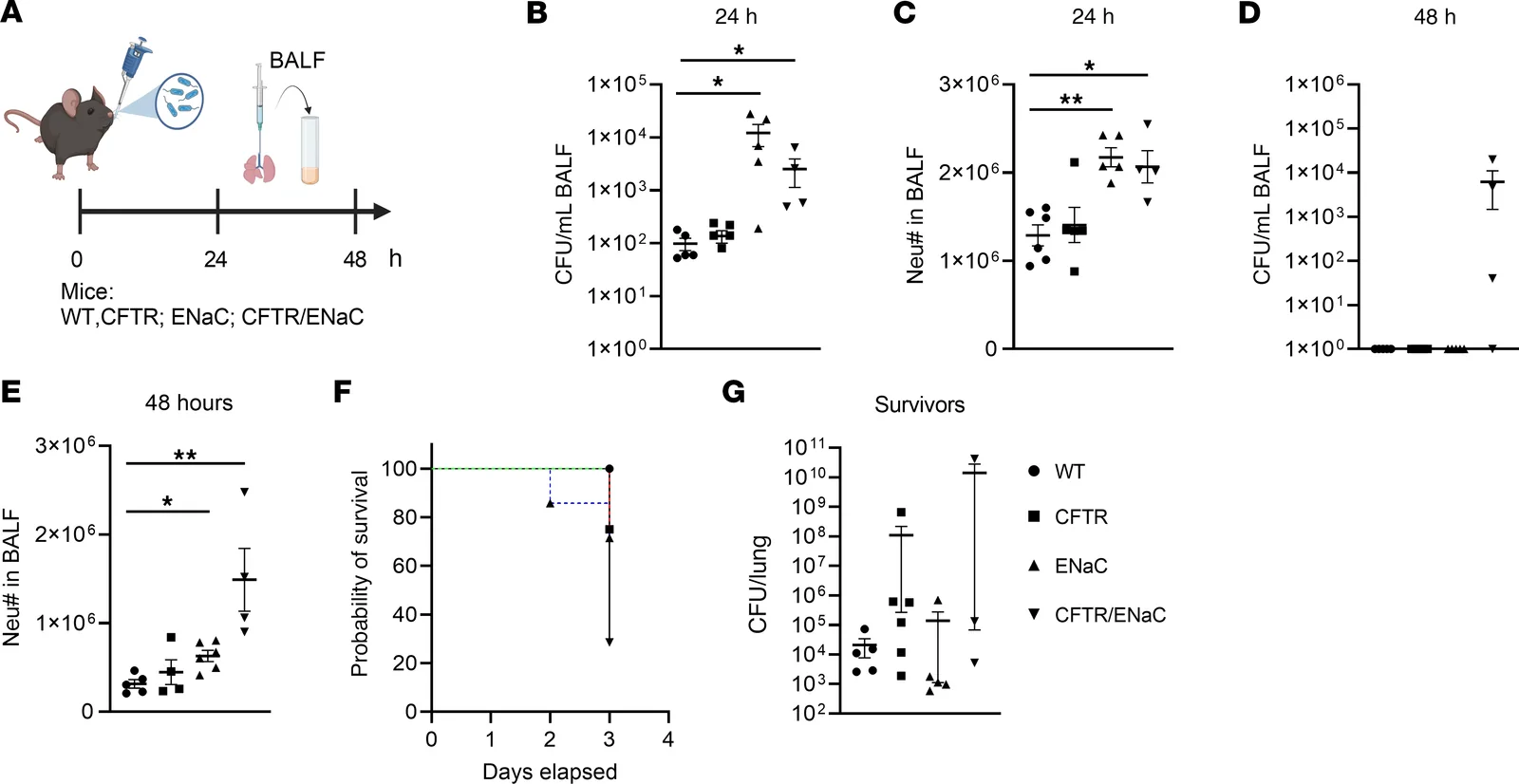
Planktonic *P. aeruginosa* fails to establish chronic lung infection in WT and CF mouse models. (**A**–**E**) WT, CFTR, ENaC, and CFTR/ENaC mice were infected with 1 × 10^6^ CFU *P*. *aeruginosa* for 24 or 48 hours. Mice were then sacrificed, and BALF was collected to assess bacterial burden and neutrophil influx. (**F** and **G**) WT, CFTR, ENaC, and CFTR/ENaC mice were infected with 4 × 10^6^ CFU *P*. *aeruginosa* for 72 hours. Mice that reached humane endpoints were sacrificed and recorded as deaths. Survival (**F**) and lung bacterial burden in surviving mice (**G**) were quantified. **A** was created in BioRender. Qin, W. (2026) https://BioRender.com/0ki5t78 (**B**–**E** and **G**) Data are presented as mean ± SEM, with individual dots representing individual mice from 1 representative experiment. Statistical significance was determined by 1-way ANOVA followed by Tukey’s multiple comparisons test; *P < 0.05, **P < 0.01. (**F**) Survival curves were analyzed using the log-rank (Mantel-Cox) test. Independent experiments yielded similar results and are representative of ≥2 experiments.

**Figure 3:**
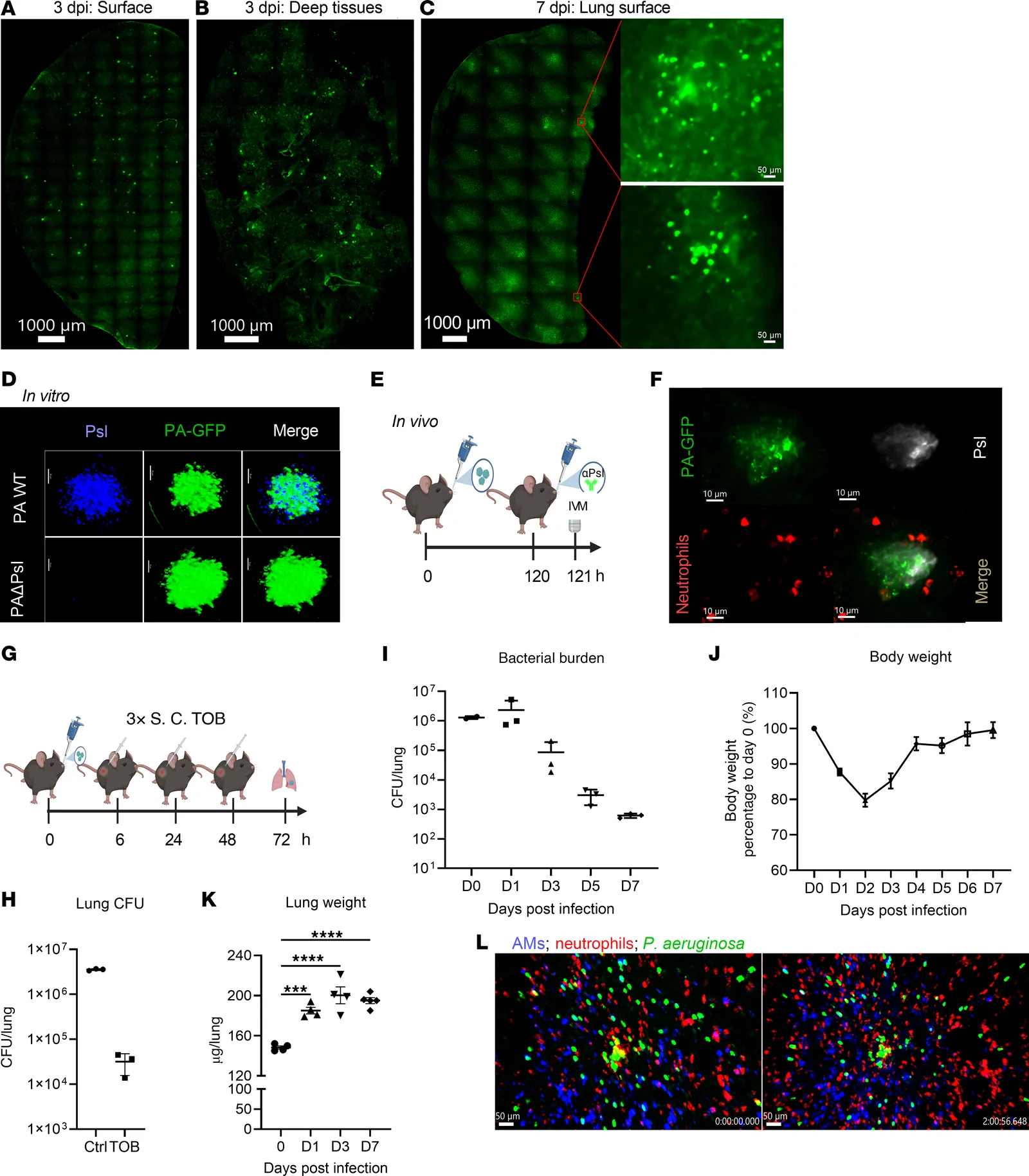
Establishment of a chronic *P. aeruginosa* infection model. WT mice were infected with *P*. *aeruginosa* agarose beads. The distribution of *P*. *aeruginosa* beads (shown in bright green) imaged by confocal microscope from the lung surface (**A**) and within deep lung tissues (**B**) at 3 days postinfection (dpi). Scale bars: 1,000 μm. (**C**) Detecting of *P*. *aeruginosa* beads (bright green) by confocal microscope imaged from the lung surface at 7 dpi. Magnified insets highlight the detailed structure of bacterial beads. Magnification: 1,000 μm. Scale bar (inserts): 50 μm. (**D**) Immunostaining of biofilm matrix polysaccharide Psl (blue) on GFP-expressing *P*. *aeruginosa* agarose beads (green) in vitro. Psl-deficient bacterial beads were used as controls to determine the specificity of the Psl antibody. Magnification: 30 μm. (**E** and **F**) Catchup mice, in which neutrophils express tdTomato (red), were infected with GFP-expressing *P*. *aeruginosa* agarose beads (green) for 5 days, followed by intranasal administration of Alexa Fluor 647–conjugated anti-Psl antibody (white) 1 hour before lung IVM imaging to visualize biofilm formation in vivo. Scale bars: 10 μm. (**G** and **H**) Mice infected with *P*. *aeruginosa* beads received tobramycin treatment every 24 hours starting 6 hours postinfection 3 times, and bacterial burden in lung tissues was measured at 72 hours postinfection. (**I**–**K**) Mice infected with *P*. *aeruginosa* beads containing 1 × 10^6^ CFU were monitored for (**I**) bacterial burden, (**J**) body weight, and (**K**) lung weight over 7 days postinfection. (**L**) IVM imaging of AMs (blue), neutrophils (red), and *P*. *aeruginosa* (green) in lung tissue for 2 hours at 7 days postinfection with *P*. *aeruginosa* beads. The complete time-lapse sequence is provided in Supplemental Video 2. Scale bars: 50 μm. (**H**, **I**, and **K**) Data are presented as mean ± SEM, with individual dots representing individual mice from 1 representative experiment. Statistical significance was determined using an unpaired 2-tailed *t* test (**H**) or 1-way ANOVA followed by Tukey’s multiple comparisons test (**K**); ***P < 0.001, ****P < 0.0001. (**J**) Data are presented as mean ± SEM from 5 mice in 1 representative experiment. Independent experiments yielded similar results and are representative of ≥2 independent experiments. **E** and **G** were created in BioRender. Qin, W. (2026) https://BioRender.com/0ki5t78

**Figure 4:**
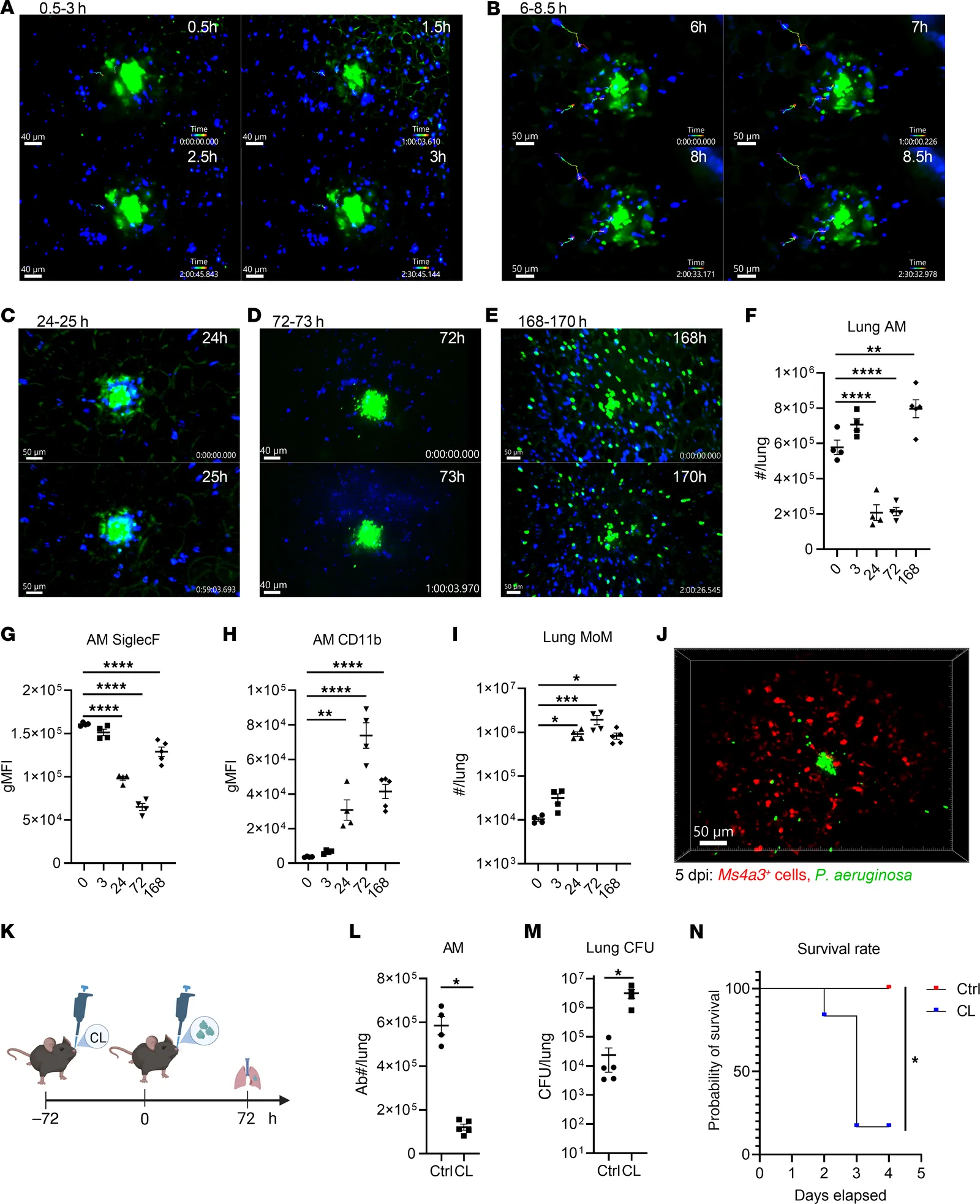
Characterization of the dynamic behavior of AMs during chronic *P. aeruginosa* infection in mice. AMs in WT mice were labeled with PKH26 at 5 days before infection with *P*. *aeruginosa* agarose beads. AM (blue) migration and *P*. *aeruginosa* beads (green) were visualized by lung IVM at 0.5 hours (**A**), 6 hours (**B**), 24 hours (**C**), 72 hours (**D**), and 168 hours (**E**) postinfection. Panel **E** focuses on the interaction between AMs and *P*. *aeruginosa*–containing beads at 7 days postinfection and was adapted from Figure 3L. The lines in panels **A** and **B** trace representative examples of AM migration paths. (**F**–**I**) Flow cytometric analysis of AM numbers (**F**), surface expression of Siglec-F (**G**) and CD11b (**H**), and enumeration of monocyte-derived macrophages (Lung MoM) (**I**) in lung tissues. (**J**) *Ms4a3-CreERT Ai14* mice were administered tamoxifen daily by intraperitoneal injection for 4 consecutive days before infection with *P*. *aeruginosa* beads. *Ms4a3*^+^ cells (red) and *P*. *aeruginosa* beads (green) in the lung tissues were visualized by IVM at 5 days postinfection (dpi). Mice were treated with clodronate liposomes (CL) prior to infection with *P*. *aeruginosa* beads (**K**). AM numbers (**L**) and bacterial burden (**M**) were quantified at the time of infection and at 3 days postinfection, respectively. Mouse survival was monitored following infection (**N**). (**F**–**I**, **L**, and **M**) Data are presented as mean ± SEM, with individual dots representing individual mice from 1 representative experiment. Statistical significance was determined using 1-way ANOVA followed by Tukey’s multiple comparisons test (**F**–**I**) or an unpaired *t* test (**L** and **M**); *P < 0.05, **P < 0.01, ***P < 0.001, ****P < 0.0001. (**N**) Survival curves were analyzed using the log-rank (Mantel-Cox) test. Independent experiments yielded similar results and are representative of ≥2 independent experiments. (**K**) was created in BioRender. Qin, W. (2026) https://BioRender.com/0ki5t78

**Figure 5:**
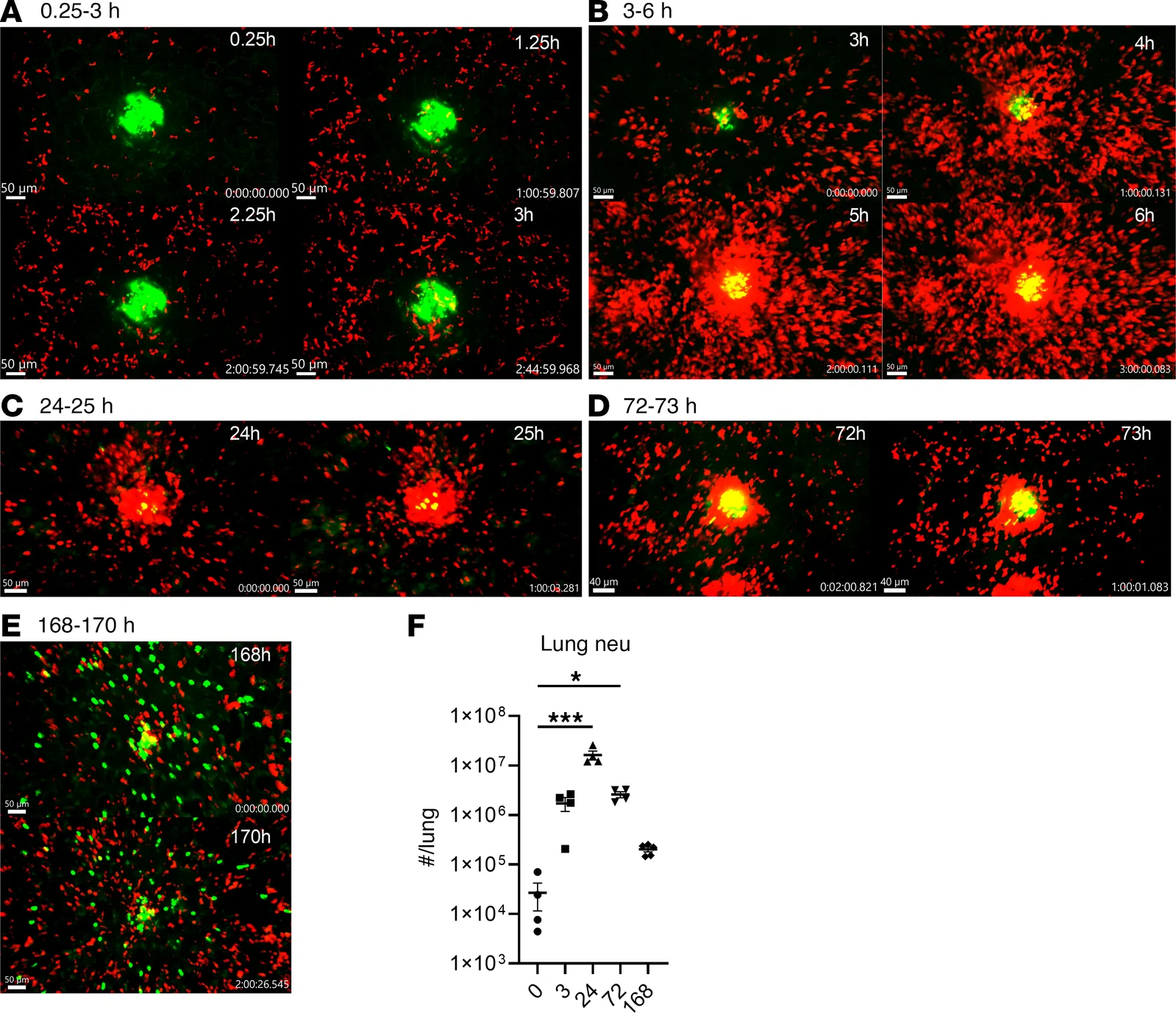
Characterization of the dynamic behavior of neutrophils during chronic *P. aeruginosa* infection in mice. Catchup mice were infected with *P. aeruginosa* agarose beads. Neutrophil migration (red) and *P*. *aeruginosa* beads (green) were visualized in the lungs by intravital microscopy (IVM) at 15 minutes (**A**), 3 hours (**B**), 24 hours (**C**), 72 hours (**D**), and 168 hours (**E**) postinfection. Panel **E** focuses on the interaction between neutrophils and *P*. *aeruginosa*–containing beads at 7 days postinfection and was adapted from Figure 3L. (**F**) Neutrophil numbers in lung tissues were quantified by flow cytometry. Data are presented as mean ± SEM. Statistical significance: *P < 0.05, ***P < 0.001. One-way ANOVA with Tukey`s post hoc test. Each data point represents an individual mouse from 1 representative experiment. Independent experiments yielded similar results and are representative of *N* ≥ 2 experiments.

**Figure 6:**
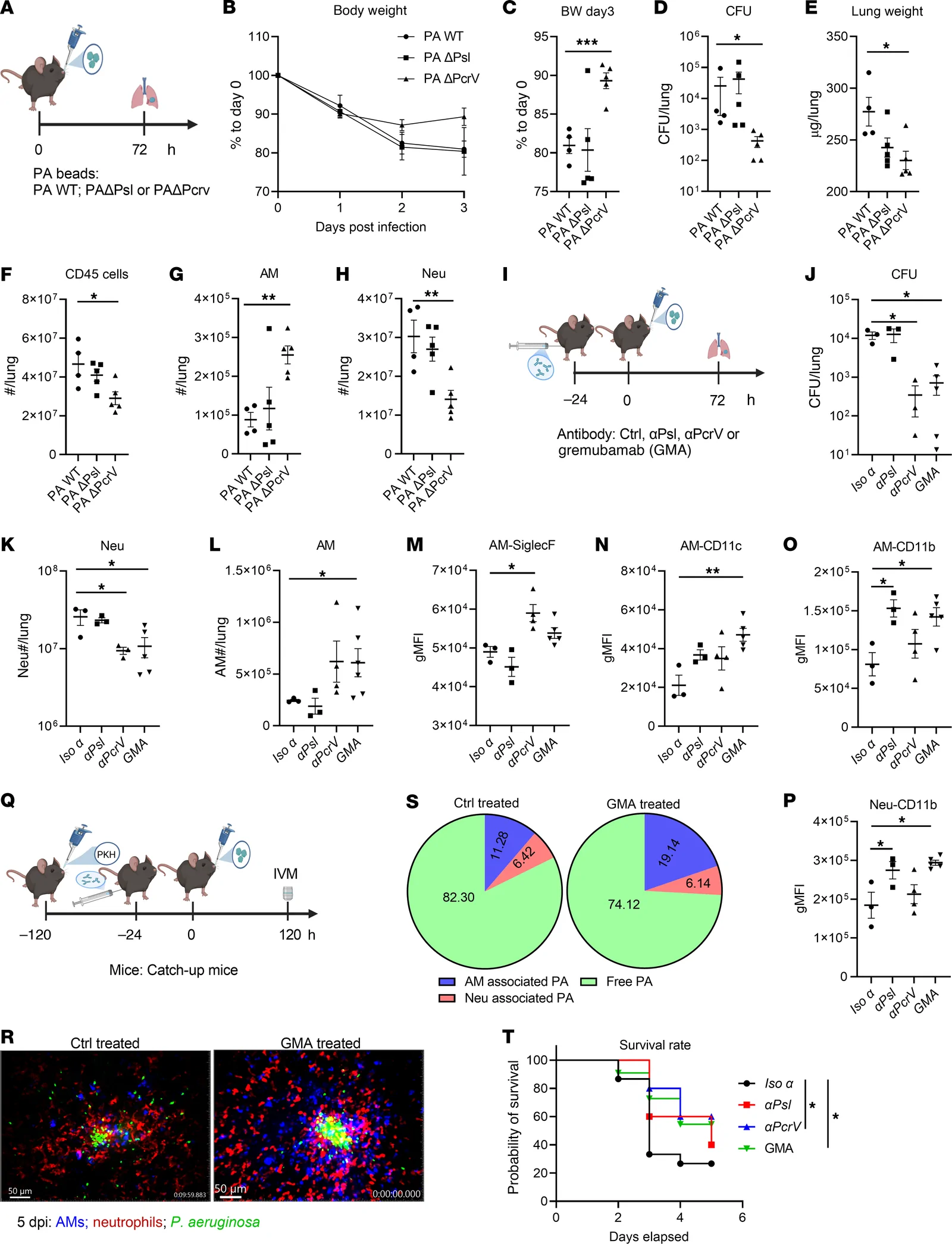
The role of Psl and PcrV in chronic *P. aeruginosa* infection in mice. (**A**) WT mice were infected with bacterial beads containing WT *P*. *aeruginosa* (PA WT), ΔPsl mutant (PAΔPsl), or ΔPcrV mutant (PAΔPcrv). Mice were then sacrificed at 72 hours postinfection. Changes in body weight over 3 days (**B**) and body weight loss at day 3 (**C**). Bacterial burden in lung tissues (**D**) and lung weight (**E**) at 72 hours postinfection. (**F**–**H**) Flow cytometric quantification of total CD45^+^ immune cells, AMs, and neutrophils (Neu) in lung tissues. (**I**) Mice were treated with control IgG, anti-Psl, anti-PcrV, or gremubamab antibody (GMA) via intraperitoneal injection 24 hours before infection with *P*. *aeruginosa* beads and sacrificed at 72 hours postinfection. Quantification of bacterial load (**J**), total Neu numbers (**K**), and AM numbers (**L**) in lung tissues. The expression of Siglec-F (**M**), CD11c (**N**), and CD11b (**O**) on AMs and CD11b expression on neutrophils (**P**). (**Q**) AMs in Catchup mice were labeled with CellVue Claret Far Red Fluorescent dye (PKH) 5 days before infection and administered GMA intraperitoneally 1 day prior to infection with GFP-expressing *P*. *aeruginosa* beads. Lung tissues were imaged by IVM at 5 days postinfection. (**R**) Representative IVM image of lung tissue at 5 days postinfection showing AMs (blue), Neu (red), and *P*. *aeruginosa* beads or individual bacteria (green). Scale bar: 50 �m. (**S**) Quantification of the proportion of individual bacteria unassociated or associated with AMs or neutrophils. Data are presented as mean ± SEM. (**T**) Survival curves of mice infected with 2 × 10^6^ CFU *P*. *aeruginosa* beads and treated with control IgG, anti-Psl, anti-PcrV, or GMA antibody. (**B** and **S**) Data are presented as mean ± SEM from 4 mice in 1 representative experiment. (**C**–**H** and **J**–**P**) Data are presented as mean ± SEM, with individual dots representing individual mice from 1 representative experiment. Statistical significance was determined using 1-way ANOVA followed by Tukey’s multiple comparisons test; *P < 0.05; **P < 0.01; ***P < 0.001. (**T**) Survival curves were analyzed using the log-rank (Mantel-Cox) test. Independent experiments yielded similar results and are representative of ≥2 independent experiments. **A**, **I**, and **Q** were created in BioRender. Qin, W. (2026) https://BioRender.com/0ki5t78

**Figure 7:**
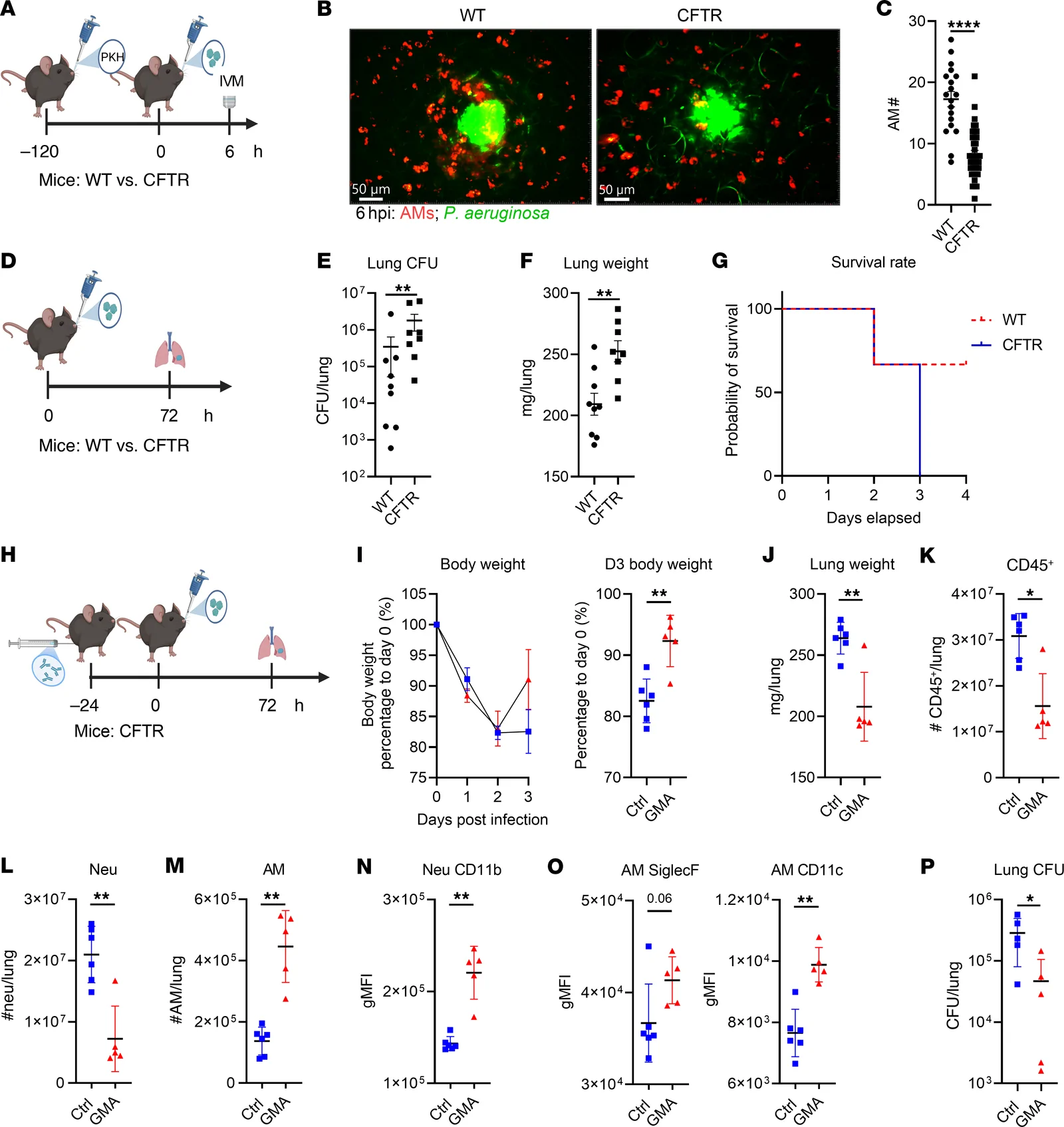
CFTR deficiency compromises defense against chronic *P. aeruginosa* infection, and gremubamab confers protection. (**A**) AMs in WT and CFTR-deficient mice were labeled with PKH26 at 5 days prior to infection with *P*. *aeruginosa* agarose beads. Lung IVM was performed at 6 hours postinfection (hpi). (**B**) Representative IVM images showing AMs (red) and *P*. *aeruginosa* beads (green). Scale bar: 50 �m. (**C**) Quantification of AMs surrounding a single bacterial bead in the lungs of WT and CFTR mice at 6 hpi. (**D**) WT and CFTR mice were infected with *P*. *aeruginosa* beads for 72 hours, after which lungs were collected for analysis. (**E**) Lung bacterial burden, (**F**) lung weight, and (**G**) survival rate. (**H**) CFTR mice were treated with gremubamab (GMA) or an isotype control antibody 24 hours prior to *P*. *aeruginosa* bead infection and analyzed 72 hours later. (**I**) Changes in body weight over 3 days (left) and body weight loss at day 3 (right). (**J**) Lung weight, (**K**) total CD45^+^ immune cells, (**L**) Neu, and (**M**) AMs in lung tissues were quantified. (**N**) Surface CD11b expression on lung neutrophils and (**O**) Siglec-F and CD11c expression on AMs were evaluated by flow cytometry. (**P**) Lung bacterial burden at 72 hpi. (**C**) Data are presented as mean ± SEM, with individual dots representing the number of AMs surrounding individual bacterial beads imaged from 5 mice in 1 representative experiment. (**E** and **F**, right panel of **I**, and **J**–**P**) Data are presented as mean ± SEM, with individual dots representing individual mice from 1 representative experiment. Statistical significance was determined using an unpaired *t* test; *P < 0.05; **P < 0.01. (**G**) Survival curves were analyzed using the log-rank (Mantel-Cox) test. (**I**, left panel) Data are presented as mean ± SEM from 4 mice in 1 representative experiment. Independent experiments yielded similar results and are representative of ≥2 independent experiments. **A**, **D**, and **H** were created in BioRender. Qin, W. (2026) https://BioRender.com/0ki5t78

**Figure 8:**
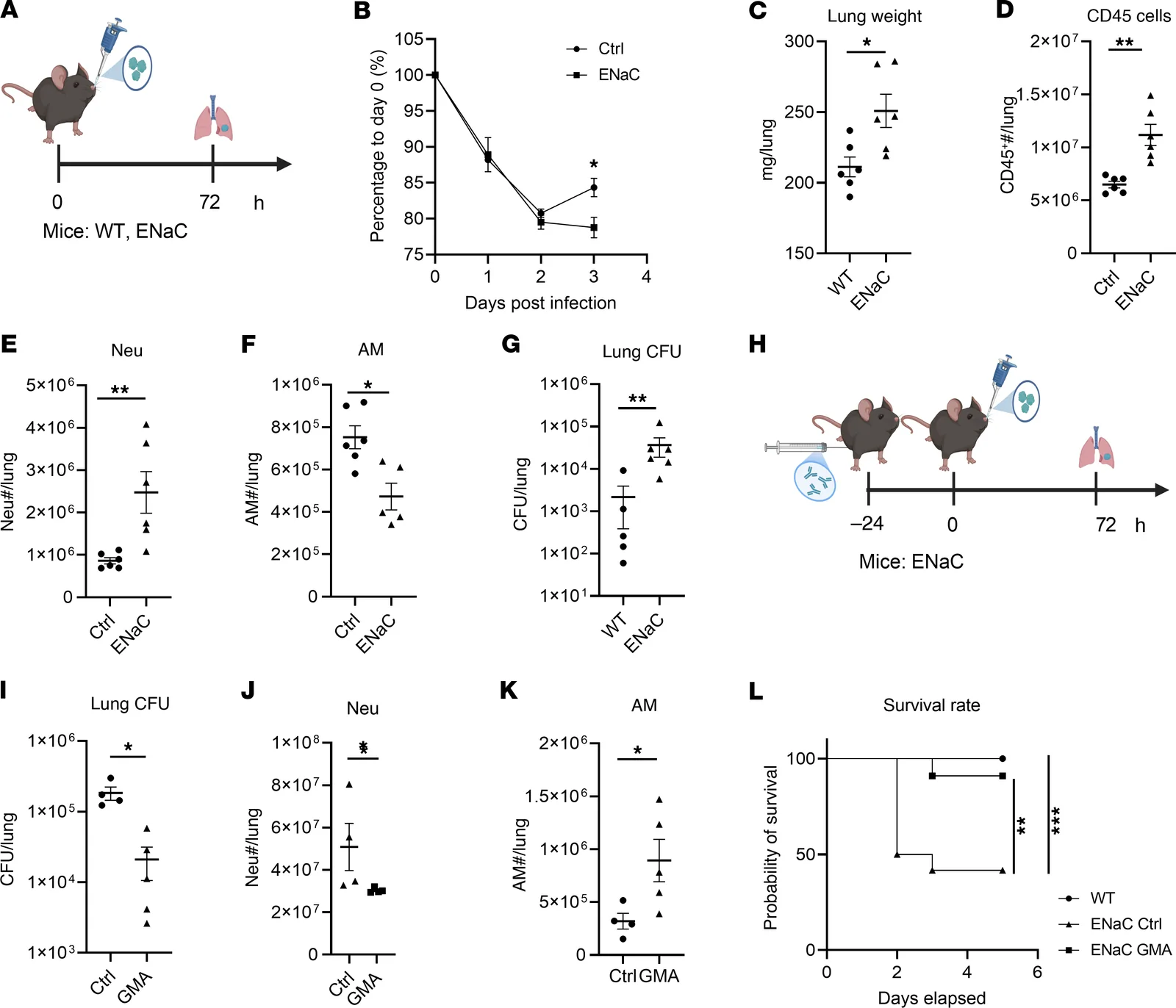
ENaC mice exhibit increased susceptibility to chronic *P.* *aeruginosa* infection and improved outcomes following gremubamab treatment. (**A**) WT and ENaC mice were infected with *P*. *aeruginosa*–laden agarose beads and analyzed 72 hours postinfection. (**B**) Changes in body weight over 3 days. (**C**) Lung weight, (**D**) total CD45^+^ immune cell numbers, (**E**) neutrophils (Neu), and (**F**) AMs in lung tissues at 72 hours postinfection. (**G**) Bacterial burden in the lungs. (**H**) ENaC mice were treated with gremubamab (GMA) or an isotype control antibody 24 hours prior to infection and analyzed 72 hours later. (**I**) Lung bacterial burden, (**J**) Neu, and (**K**) AMs were quantified. (**L**) Survival rate of mice following infection with a higher dose of *P*. *aeruginosa* beads (5 × 10^5^ CFU). (**B**) Data are presented as mean ± SEM from 3 mice in 1 representative experiment. (**C**–**G** and **I**–**K**) Data are presented as mean ± SEM, with individual dots representing individual mice from 1 representative experiment. Statistical significance was determined using an unpaired *t* test. (**L**) Survival curves were analyzed using the log-rank (Mantel-Cox) test; *P < 0.05; **P < 0.01, ***P < 0.001. Independent experiments yielded similar results and are representative of ≥2 independent experiments. **A** and **H** were created in BioRender. Qin, W. (2026) https://BioRender.com/0ki5t78

**Figure 9:**
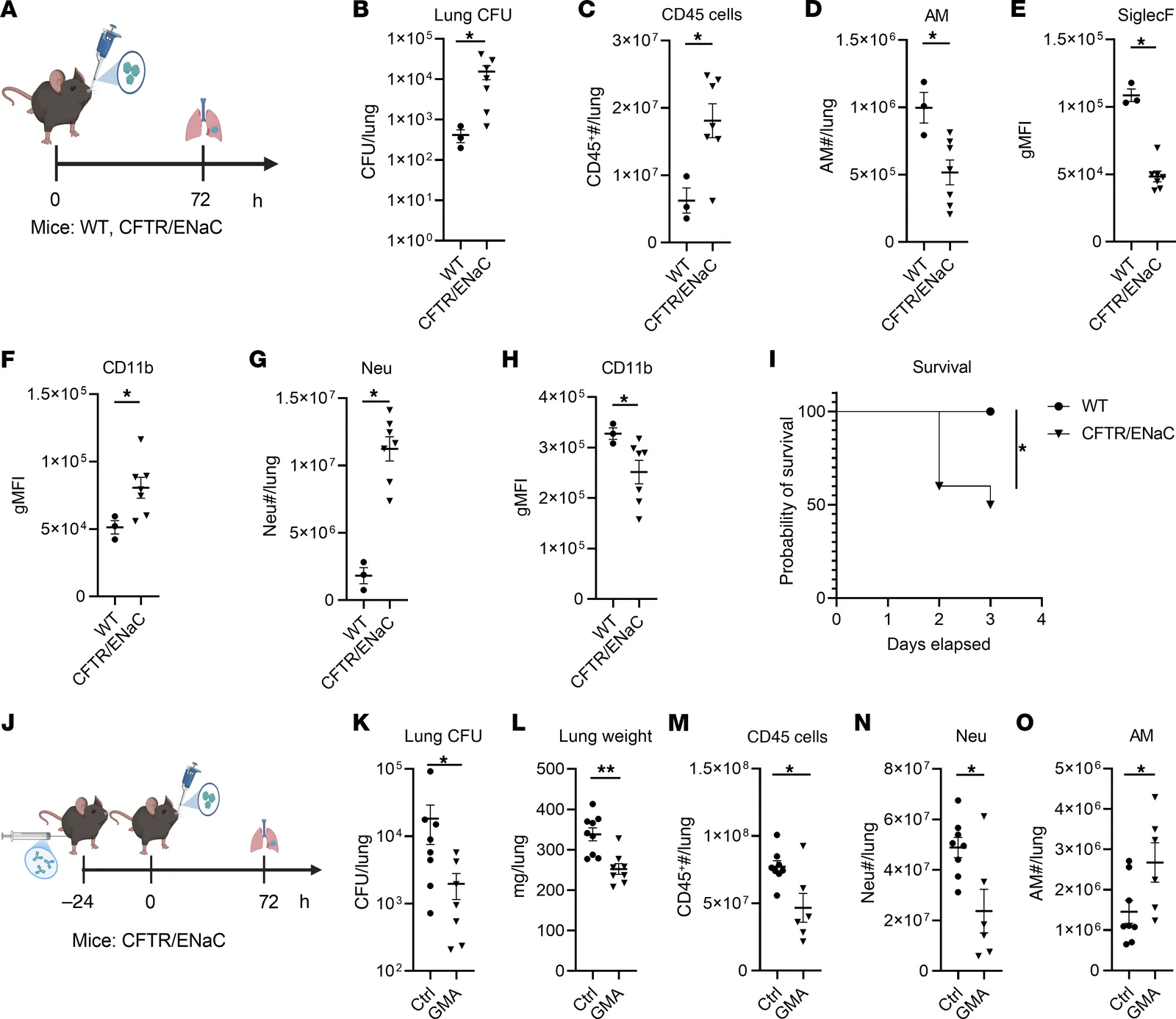
CFTR/ENaC mice exhibit increased susceptibility to chronic *P.* *aeruginosa* infection and improved outcomes following gremubamab treatment. (**A**) WT and CFTR/ENaC mice were infected with *P*. *aeruginosa* agarose beads and analyzed 72 hours postinfection. (**B**) Lung bacterial burden was quantified. (**C**–**H**) Flow cytometric analysis of total CD45^+^ immune cells (**C**), AMs (**D**) and their surface expression of Siglec-F (**E**) and CD11b (**F**), as well as neutrophils (Neu; **G**) and their surface CD11b expression (**H**). (**I**) Survival rate of mice following infection with a higher dose of *P*. *aeruginosa* beads (5 × 10^5^ CFU). (**J**) CFTR/ENaC mice were treated with gremubamab or an isotype control antibody 24 hours prior to *P*. *aeruginosa* bead infection and analyzed after 72 hours. (**K**) Lung bacterial burden, (**L**) lung weight, (**M**) total CD45^+^ immune cells, (**N**) Neu, and (**O**) AMs were quantified. (**B**–**H** and **K**–**O**) Data are presented as mean ± SEM, with individual dots representing individual mice from 1 representative experiment. Statistical significance was determined using an unpaired *t* test. (**L**) Survival curves were analyzed using the log-rank (Mantel-Cox) test; *P < 0.05; **P < 0.01. Independent experiments yielded similar results and are representative of ≥2 independent experiments. **A** and **J** were created in BioRender. Qin, W. (2026) https://BioRender.com/0ki5t78

**Figure 10:**
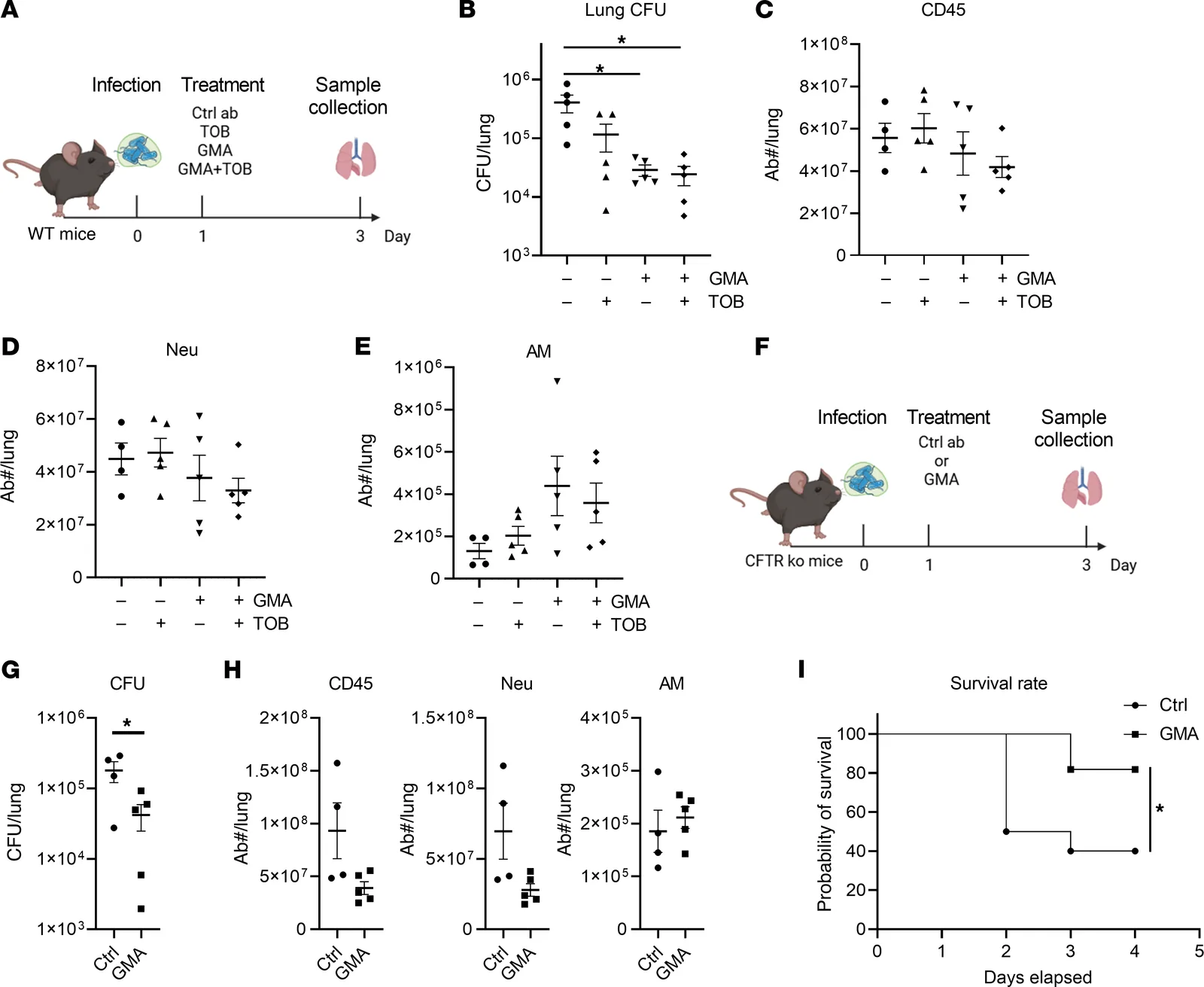
Therapeutic administration of gremubamab after establishment of *P.* *aeruginosa* infection reduces bacterial burden and improves survival. (**A**) WT mice were infected with *P*. *aeruginosa* beads and treated 24 hours later with a control antibody, gremubamab (GMA), tobramycin (TOB), or a combination of GMA and TOB. (**B**–**E**) Bacterial burden and the numbers of total leukocytes (CD45^+^ cells), neutrophils (Neu), and AMs in lung tissues were quantified 3 days after infection. (**F**) CFTR mice were infected with *P*. *aeruginosa* beads and treated 24 hours later with either a control antibody or GMA. (**G** and **H**) Bacterial burden and the numbers of total leukocytes (CD45^+^ cells), Neu, and AMs in lung tissues were quantified 3 days after infection. (**I**) Survival of CFTR mice treated with Ctrl or GMA antibodies following infection with a higher dose of *P*. *aeruginosa* beads. (**B**–**E**, **G**, and **H**) Data are presented as mean ± SEM, with individual dots representing individual mice from 1 representative experiment. Statistical significance was determined using 1-way ANOVA followed by Tukey’s multiple comparisons test (**B**–**E**) or an unpaired *t* test (**G** and **H**). (**I**) Survival curves were analyzed using the log-rank (Mantel-Cox) test. *P < 0.05. Independent experiments yielded similar results and are representative of ≥2 independent experiments. **A** and **F** were created in BioRender. Qin, W. (n.d.) https://BioRender.com/yajdzlp

## Version 1 09/22/2026

In-Press Preview

## Version 2 11/16/2026

Electronic publication
