# Supplementary material for: Targeting virulence factors Psl and PcrV restores host defense in bronchiectasis and cystic fibrosis models
Source: J Clin Invest. 2026 Sep 22;136(22):e205523. doi: 10.1172/JCI205523 (PMC13767668; doi:10.1172/JCI205523)
Supplement: Supplementary file 1 [file jci-136-205523-s037.pdf]

Supplementary materials

Supplemental Table： Flow cytometry antibodies

Antibody	Clone	Source	Catalog	Dilution
Purified anti-CD16/32	93	Biolegend	Cat# 101302; Lot#B298973	200
GhostRed 710		Tonbiosciences	Cat# 130871-T100	1000
Ly6C (PercpCy5.5)	HK1.4	BD Biosciences	Cat#45-5932-82, Lot#2309273	100
CD45 (BV510)	30-F11	Biolegend	Cat#103138; Lot# B386738	200
CD45 (PE)	30-F11	BD Biosciences	Cat#12-0451-82	200
CD11c （BUV805)	HL3	BD Biosciences	Cat#749090	200
Siglec-F	E50-2440	BD biosciences	Cat#565934	200
Ly-6G （BV650）	1A8	BD Biosciences	Cat#740554	200
CD64 (AF647)	X54-5/7	BD Biosciences	Cat#558539	100
CD11b (PE-Cy7)	M1/70	BD Biosciences	Cat#25-0112-82	400
CD19 (eFluor 450)	eBio1D3	BD Biosciences	Cat#48-0193-82	100
CD3 (APC)	17A2	BD Biosciences	Cat#17-0032-82	100

Supplementary figure 1

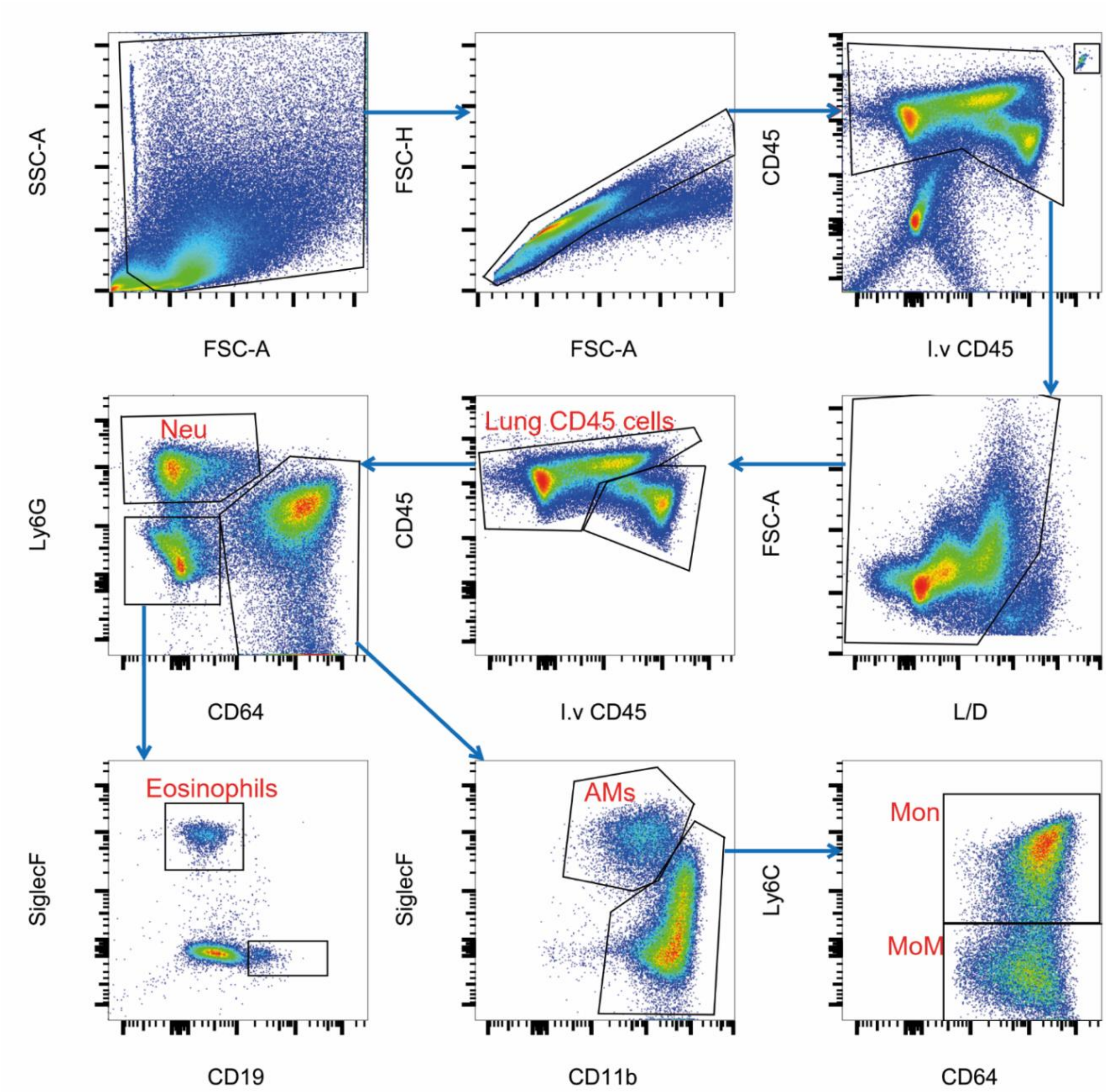

Figure S1. Flow cytometric gating strategy for lung immune cell populations.

Representative flow cytometry plots showing the sequential gating strategy used to identify lung immune cell subsets in mice infected with *P. aeruginosa* beads. After exclusion of debris (FSC-A vs SSC-A), doublets (FSC-H vs FSC-A), and dead cells (L/D), $CD45^+$ leukocytes were selected and further discriminated into intravascular (i.v. $CD45^+$) and lung-resident $CD45^+$ cells. Lung $CD45^+$ cells were subsequently gated to identify neutrophils ($Ly6G^+CD64^-$), eosinophils ($SiglecF^+CD64^-$), alveolar macrophages (AMs; $CD64^+SiglecF^+CD11b^{lo}$), and monocytes (Mon; $SiglecF^-Ly6C^+CD64^+$) and monocyte-derived macrophages (MoM, $SiglecF^-Ly6C^-CD64^+$). Gates are representative of those used for all analyses.

Supplementary figure 2

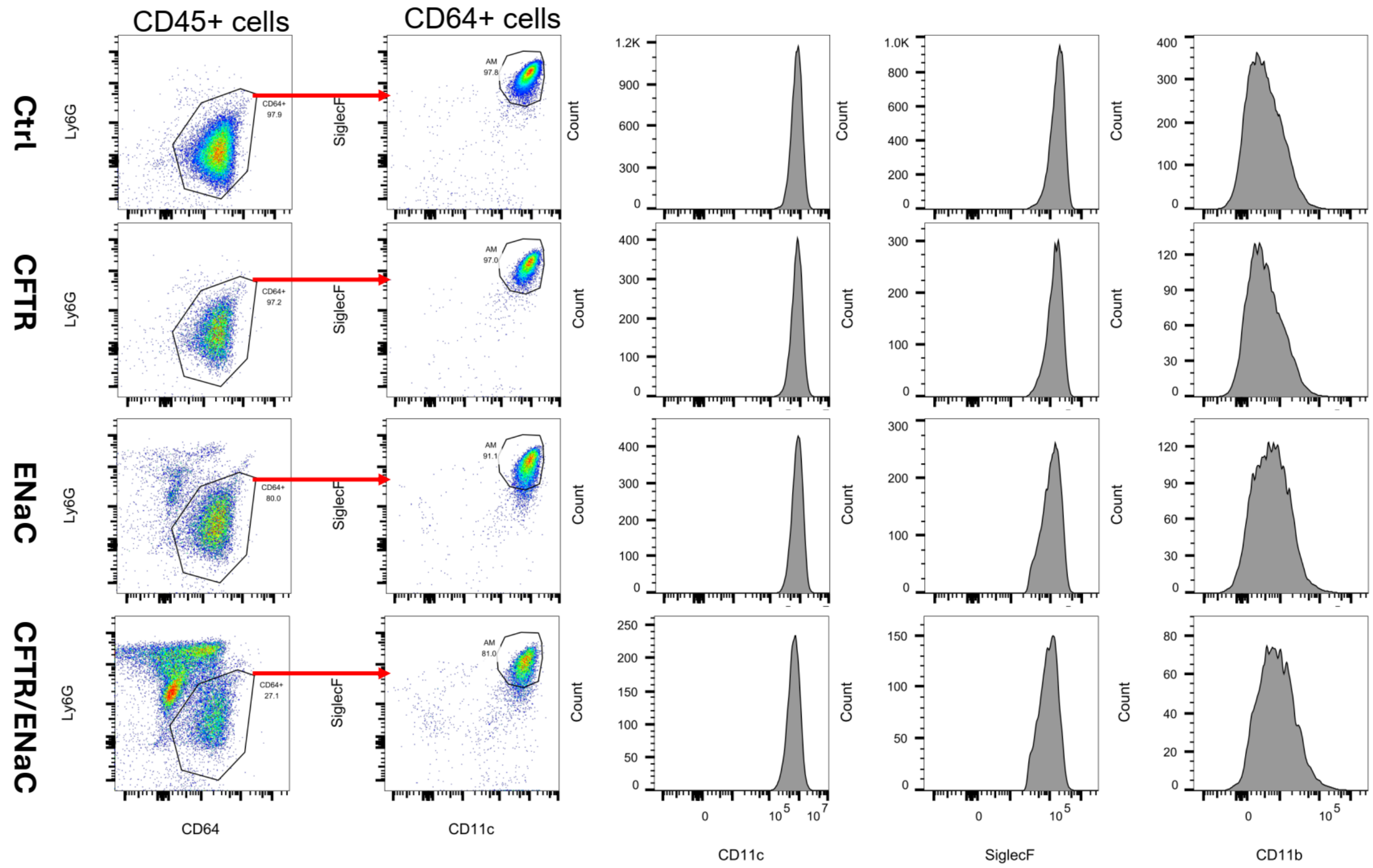

Figure S2. Flow cytometric identification of alveolar macrophages in cystic fibrosis (CF) mouse models.

Representative flow cytometry plots showing the sequential gating strategy used to identify alveolar macrophages (AMs; CD64^+Siglec-F^highCD11c^high) in bronchoalveolar lavage fluid collected from CF mouse models. Histograms showing the expression of CD11c, Siglec-F, and CD11b on gated AMs are presented in the right panels.

Supplementary figure 3

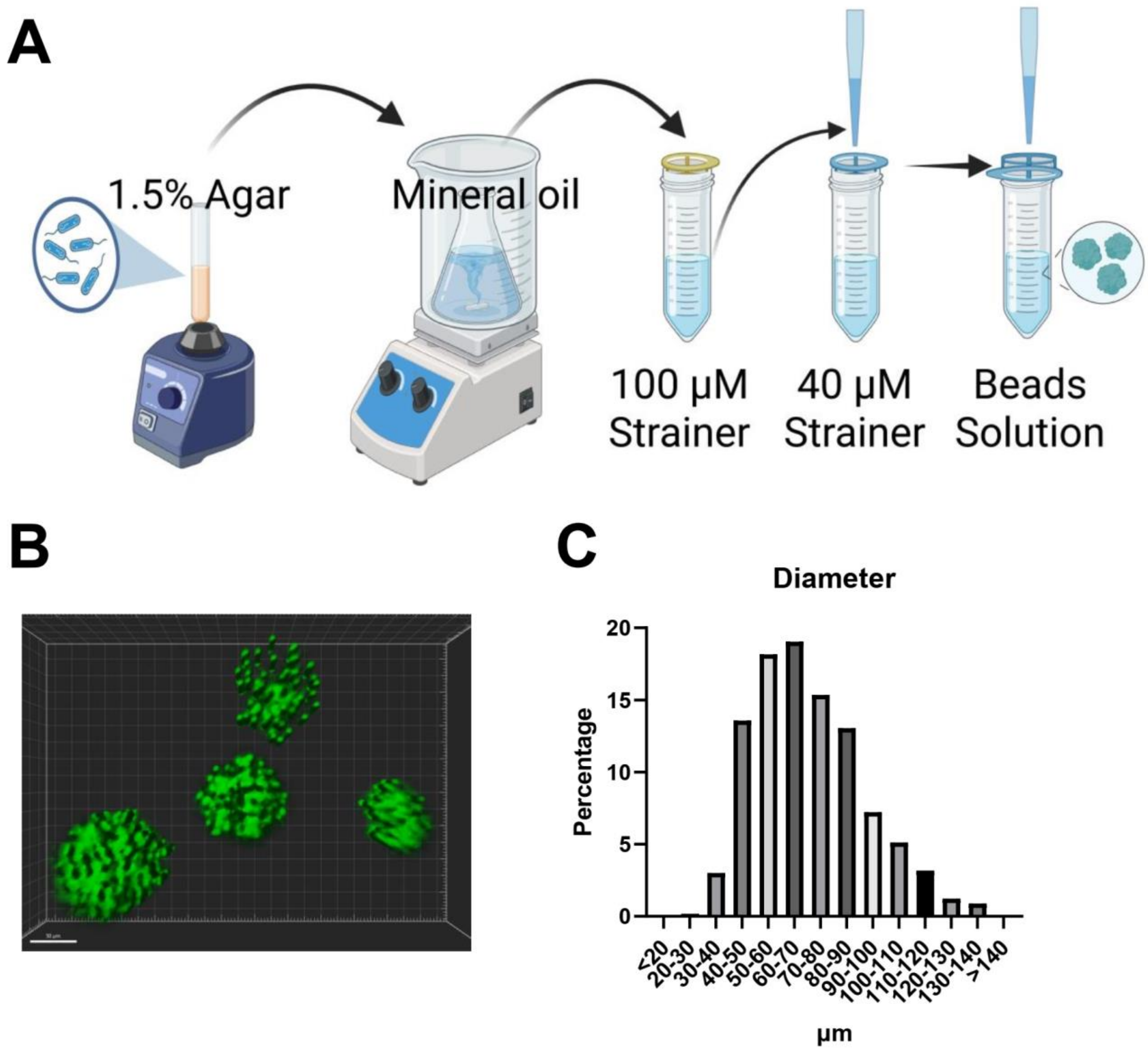

Figure S3. Preparation of *Pseudomonas aeruginosa* agarose beads.

A. Schematic of agarose bead preparation. *Pseudomonas aeruginosa* suspended in BHI medium containing 1.5% agarose was emulsified in mineral oil, mixed on ice for 30 minutes, and sequentially filtered through 100 µm and 40 µm strainers. Bacterial agarose beads retained on the 40 µm strainer were washed off with cold PBS. Created in BioRender. Qin, W. (2026) https://BioRender.com/ojm6nun.

B. Representative 3D confocal image of fluorescent agarose beads. Scale bar, 100 µm.

C. Size distribution of agarose beads, shown as the percentage of beads across diameter ranges (µm).

Supplementary figure 4

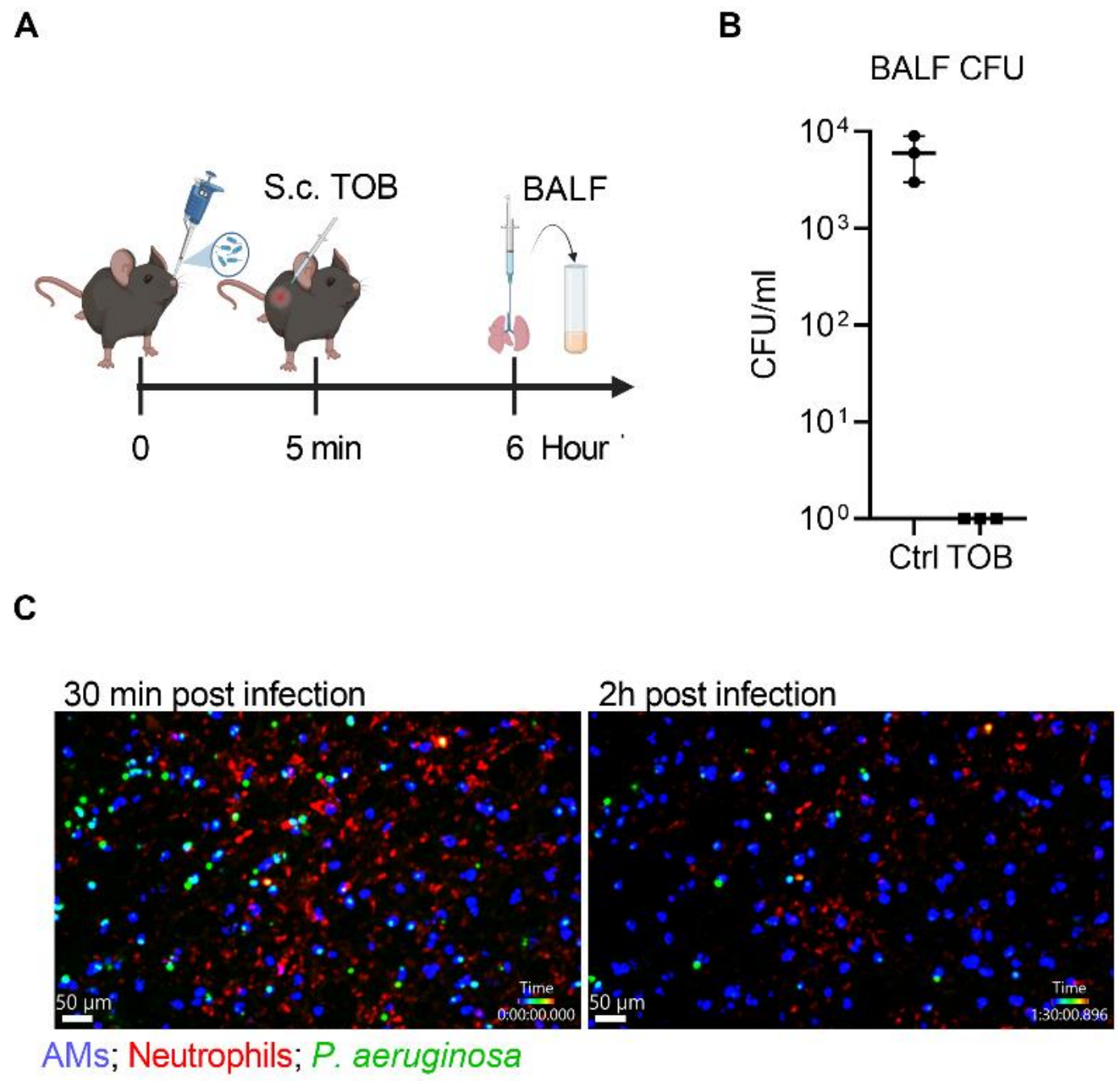

Figure S4. Tobramycin treatment and intravital imaging of mice infected with planktonic *Pseudomonas aeruginosa*.

A–B. Mice were infected with planktonic *Pseudomonas aeruginosa* and treated subcutaneously (s.c.) with tobramycin 5 minutes after infection. Pulmonary bacterial burden was quantified 6 hours post-infection. (B)Data are presented as mean ± SEM, with individual dots representing individual mice from one representative experiment. Independent experiments yielded similar results and are representative of ≥2 independent experiments.

C. Mice were infected with GFP-expressing planktonic *Pseudomonas aeruginosa*. Intravital imaging was performed from 30 minutes to 2 hours post-infection to visualize bacterial phagocytosis by alveolar macrophages (blue) and neutrophils (red).

Supplementary figure 5

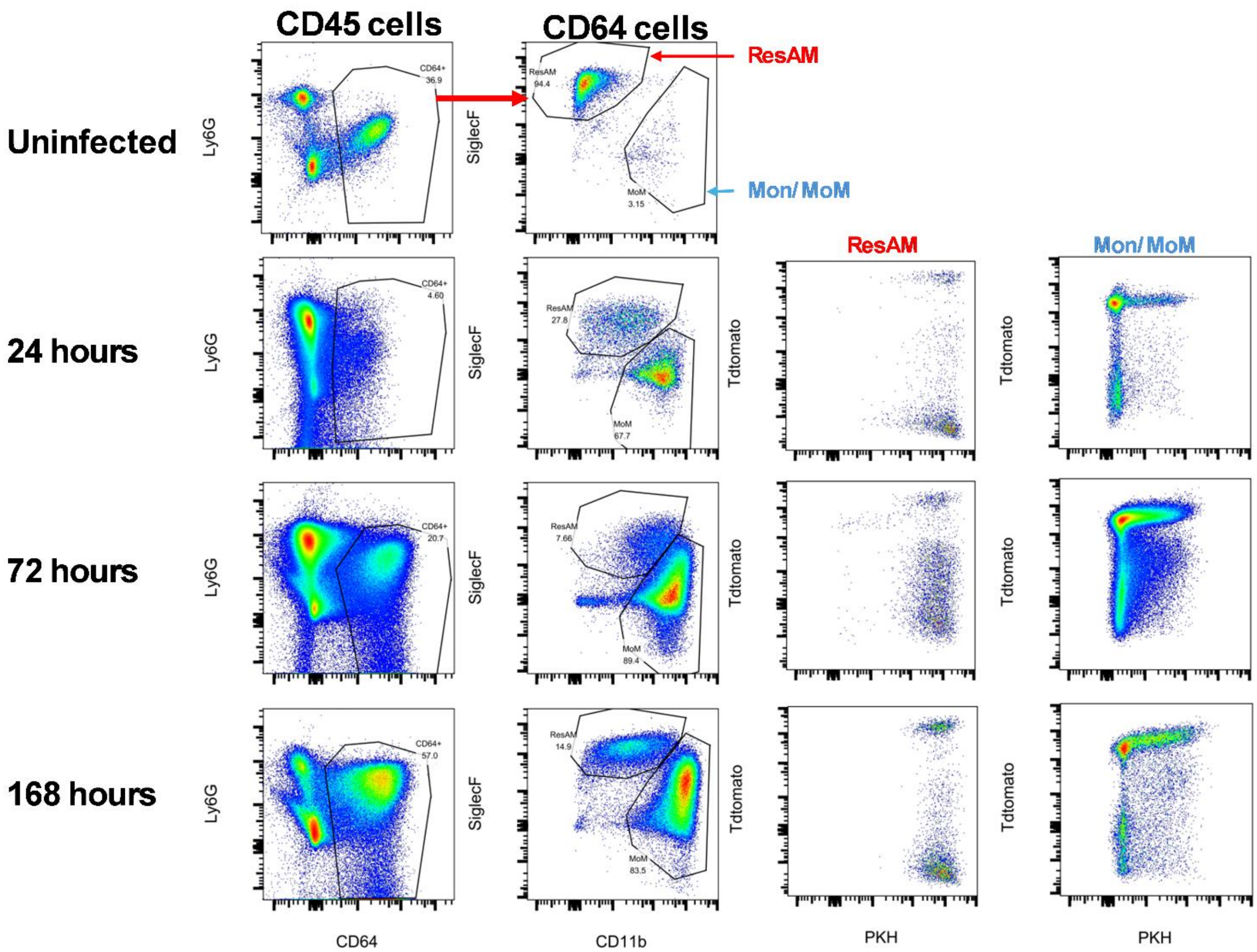

Figure S5. Distinguish resident alveolar macrophages from monocyte-derived macrophages.

Ms4a3 Cre Rosa TdT mice were given PKH prior to infection (labels AMs) and followed by *P. aeruginosa* bead infection for 1, 3 or 7 days. Lung tissues were then collected for flow cytometric analysis. The Siglec-F high macrophage population consisted exclusively of PKH26-positive cells, suggesting no contribution from monocyte-derived macrophages. In contrast, the CD11b high population was predominantly tdTomato (Ms4a3)-positive and largely lacked PKH26 labeling, consistent with newly recruited monocyte-derived alveolar macrophages.

Legends for Supplementary Videos

Video S1. Phagocytosis of planktonic *Pseudomonas aeruginosa* by alveolar immune cells.

Phagocytosis of planktonic *Pseudomonas aeruginosa* (green) by alveolar macrophages (blue) and neutrophils (red) was recorded starting 30 minutes after infection with planktonic bacteria.

Video S2. Phagocytosis of *Pseudomonas aeruginosa* by alveolar macrophages and neutrophils at 7 days after infection with *Pseudomonas aeruginosa* beads.

Alveolar phagocytosis of *Pseudomonas aeruginosa* (green) by macrophages (blue) and neutrophils (red) was recorded 7 days after infection with *Pseudomonas aeruginosa* beads. Scale bar, 100 µm.

Video S3. Early migration of alveolar macrophages following *Pseudomonas aeruginosa* bead infection.

Migration of alveolar macrophages (blue) toward *Pseudomonas aeruginosa* beads (green) was recorded from 15 minutes to 3 hours after infection. Scale bar, 30 µm.

Video S4. Migration of alveolar macrophages at 6 hours after *Pseudomonas aeruginosa* bead infection.

Migration of alveolar macrophages (blue) toward *Pseudomonas aeruginosa* beads (green) was recorded from 6 to 8.5 hours after infection. Scale bar, 50 µm.

Video S5. Migration of alveolar macrophages at 24 hours after *Pseudomonas aeruginosa* bead infection.

Migration of alveolar macrophages (blue) toward *Pseudomonas aeruginosa* beads (green) was recorded from 24 to 25 hours after infection. Scale bar, 50 µm.

Video S6. Migration of alveolar macrophages at 72 hours after *Pseudomonas aeruginosa* bead infection.

Migration of alveolar macrophages (blue) toward *Pseudomonas aeruginosa* beads (green) was recorded at 72 hours after infection. Scale bar, 50 µm.

Video S7. Migration of alveolar macrophages at 168 hours after *Pseudomonas aeruginosa* bead infection.

Migration of alveolar macrophages (blue) toward *Pseudomonas aeruginosa* beads (green) was recorded at 168 hours after infection. Scale bar, 50 µm.

Video S8. Early migration of neutrophils following *Pseudomonas aeruginosa* bead infection.

Migration of neutrophils (red) toward *Pseudomonas aeruginosa* beads (green) was recorded from 15 minutes to 3 hours after infection. Scale bar, 50 µm.

Video S9. Migration of neutrophils from 3 to 6 hours after *Pseudomonas aeruginosa* bead infection.

Migration of neutrophils (red) toward *Pseudomonas aeruginosa* beads (green) was recorded from 3 to 6 hours after infection. Scale bar, 100 µm.

Video S10. Migration of neutrophils at 24 hours after *Pseudomonas aeruginosa* bead infection.

Migration of neutrophils (red) toward *Pseudomonas aeruginosa* beads (green) was recorded from 24 to 25 hours after infection. Scale bar, 100 µm.

Video S11. Migration of neutrophils at 72 hours after *Pseudomonas aeruginosa* bead infection.

Migration of neutrophils (red) toward *Pseudomonas aeruginosa* beads (green) was recorded for 1 hour at 72 hours after infection. Scale bar, 50 µm.

Video S12. Migration of neutrophils at 168 hours after *Pseudomonas aeruginosa* bead infection.

Migration of neutrophils at *Pseudomonas aeruginosa* beads (green and circled with white line) was recorded at 168 hours post-infection in Catchup mice. Alexa Fluor 488–conjugated anti-Ly6G (clone 1A8) was injected intravenously via the tail vein 10 minutes after the start of recording to distinguish neutrophils within lung tissue (red) from those remaining in the vasculature (yellow). Scale bar, 100 µm.
